# Enhanced gorilla troops optimizer powered by marine predator algorithm: global optimization and engineering design

**DOI:** 10.1038/s41598-024-57098-8

**Published:** 2024-04-01

**Authors:** Mohamed H. Hassan, Salah Kamel, Ali Wagdy Mohamed

**Affiliations:** 1 Department of Electrical Engineering, Faculty of Engineering, Aswan University, Aswan, 81542 Egypt; 2 Operations Research Department, Faculty of Graduate Studies for Statistical Research, Cairo University, Giza, 12613 Egypt

**Keywords:** Gorilla troops optimizer, Marine predator algorithm, CEC2019, Swarm intelligence, Computer science, Computational science, Applied mathematics

## Abstract

This study presents an advanced metaheuristic approach termed the Enhanced Gorilla Troops Optimizer (EGTO), which builds upon the Marine Predators Algorithm (MPA) to enhance the search capabilities of the Gorilla Troops Optimizer (GTO). Like numerous other metaheuristic algorithms, the GTO encounters difficulties in preserving convergence accuracy and stability, notably when tackling intricate and adaptable optimization problems, especially when compared to more advanced optimization techniques. Addressing these challenges and aiming for improved performance, this paper proposes the EGTO, integrating high and low-velocity ratios inspired by the MPA. The EGTO technique effectively balances exploration and exploitation phases, achieving impressive results by utilizing fewer parameters and operations. Evaluation on a diverse array of benchmark functions, comprising 23 established functions and ten complex ones from the CEC2019 benchmark, highlights its performance. Comparative analysis against established optimization techniques reveals EGTO's superiority, consistently outperforming its counterparts such as tuna swarm optimization, grey wolf optimizer, gradient based optimizer, artificial rabbits optimization algorithm, pelican optimization algorithm, Runge Kutta optimization algorithm (RUN), and original GTO algorithms across various test functions. Furthermore, EGTO's efficacy extends to addressing seven challenging engineering design problems, encompassing three-bar truss design, compression spring design, pressure vessel design, cantilever beam design, welded beam design, speed reducer design, and gear train design. The results showcase EGTO's robust convergence rate, its adeptness in locating local/global optima, and its supremacy over alternative methodologies explored.

## Introduction

Optimization is widely recognized as the most effective approach for identifying optimal solutions within complex domains and for various real-world problems. Over the past few decades, numerous advanced optimization techniques have been developed to address a wide range of optimization challenges^1^. These techniques can be broadly classified into two categories: deterministic approaches and stochastic approaches. Deterministic approaches, including Newton's method^2^ and the conjugate gradient method^3^, typically utilize gradient information to guide the search process. While these methods are effective for functions with a single peak, they often struggle to escape local minima when confronted with complex nonlinear problems. On the other hand, metaheuristic algorithms, which fall under the category of stochastic methods, have emerged as successful alternatives to exact methods for solving real-world optimization problems^4^. Metaheuristics offer several benefits, including simplicity, problem independence, flexibility, and the ability to operate without requiring gradient information^5^. Stochastic methods treat the problem as a black box, relying solely on input and output states without the need for derivative information. This characteristic enables them to address various types of optimization problems without fundamentally altering the algorithm's main structure. One notable distinction between traditional deterministic methods and metaheuristic algorithms is that the latter typically employ multiple initial solutions. Importantly, the choice of the early solution does not significantly impact the optimization performance of the technique. This property has garnered significant attention from researchers due to its implications for optimization problem-solving. In summary, metaheuristic algorithms possess desirable qualities that make them highly appealing to researchers. These algorithms are characterized by their generality, enabling them to tackle diverse optimization problems without altering their fundamental framework. Additionally, they exhibit robustness, allowing them to operate effectively without relying on gradient information and utilizing multiple initial solutions.

**Figure 1:**
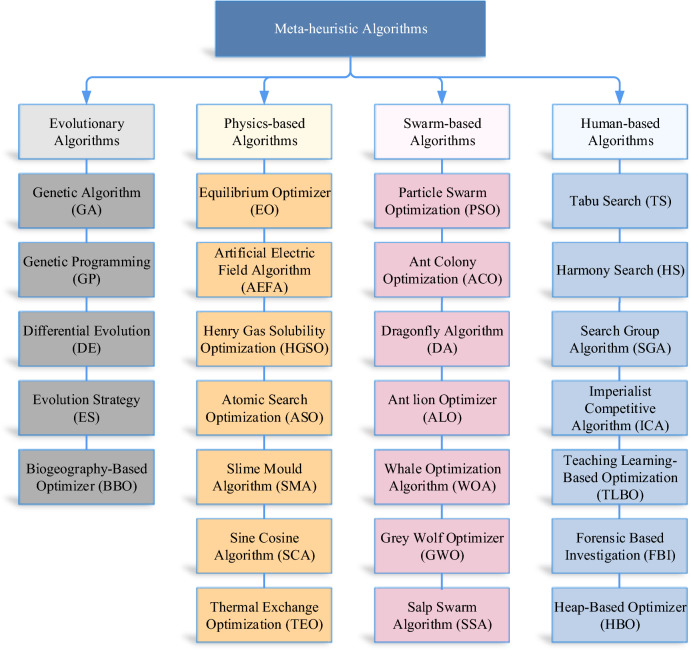
Classification of meta-heuristic algorithms.

Metaheuristic algorithms draw inspiration from various sources (as illustrated in Fig. 1), such as Human behavior, physical phenomena, swarm-based, and evolutionary concepts^6^. Evolutionary-based algorithms, such as Genetic Algorithm (GA)^7^, Differential Evolution (DE)^8^, Evolutionary Programming (EP)^9^, Starling murmuration optimizer^10^, and quantum-based avian navigation optimizer algorithm^11^, simulate Darwinian biological evolution. Physics-based algorithms, such as Equilibrium Optimizer (EO)^12^, Artificial Electric Field Algorithm (AEFA)^13^, Henry Gas Solubility Optimization (HGSO)^14^, Slime Mould Algorithm (SMA)^15^, and others, are inspired by the laws of physics. Swarm-based algorithms, including Particle Swarm Optimization (PSO)^16^, Artificial Bee Colony (ABC)^17^, and many more, simulate the collaborative behavior of biological groups in nature. Human- based algorithms, such as Tabu search (TS)^18^, Teaching–Learning-Based Optimization (TLBO)^19^, Alpine skiing optimization (ASO)^20^, and Harmony search (HS)^21^, derived from some human activities in the community. These nature-inspired metaheuristic algorithms exhibit distinct characteristics, but they all incorporate two crucial phases in the search process: exploration and exploitation^6^. During the exploration phase, the search agents endeavor to cover the entire target space extensively in order to identify areas that may contain the optimal solution. Subsequently, in the exploitation phase, more focused local searches are conducted to refine the quality and precision of the obtained optimal solution. Maintaining a suitable balance between exploration and exploitation is of paramount importance for a well-designed optimizer. By striking this balance, the algorithm can effectively navigate the search space, ensuring both comprehensive exploration and fine-grained exploitation. This balance enables the optimizer to avoid getting stuck in suboptimal solutions while efficiently converging towards the global optimum. In recent years, various optimization algorithms have been extensively utilized to address several optimization problems such as the multidisciplinary design optimization problem^22^, the semi-submersible platform boom problem^23^, and economic load dispatch in large scale power systems^24^, optimizing the artificial neural network for disease diagnosis^25^, detecting coronavirus disease 2019 (COVID-19) disease^26,27^.

It is important to note that the No Free Lunch (NFL) theorem asserts that no single technique can perform optimally on all problems^28–30^. This motivates academics to suggest new techniques or enhance present ones to address specific optimization challenges. Each algorithm possesses its own strengths and weaknesses, and applying them to different real-world problems can lead to improved results.

One noteworthy algorithm is the Gorilla Troops Optimizer (GTO)^31^, which has demonstrated superior performance and has been applied in various fields. For instance, it has been used in parameter estimation of solar PV cells^32^, feature selection for biomedical data^33^, the probabilistic optimal integration of renewable distributed generators considering the time-varying load^34^, optimal power flow (OPF) integrating thyristor-controlled series capacitors^35^, optimal parameters extracting of fuel cell^36^, automated settings of overcurrent relays considering transformer phase shift and distributed generators^37^, and more. Despite its successes, GTO still has certain limitations, such as imbalanced exploration and exploitation, a tendency to get trapped in local optima during late iterations, and stronger exploitation capabilities than exploration capabilities. Therefore, many scholars have begun updating and enhancing the performance of the GTO algorithm and applied it to solve many optimization problems and to generate higher-quality solutions from different aspects such as hybrid arithmetic optimization algorithm and GTO (AOA-GTO) algorithm for Micro-Robotics Systems^38^, opposition-based learning and parallel strategies GTO (OPGTO) for 3D node localization of wireless sensor networks^39^, Quantum GTO (QGTO) for the optimum tuning of power system stabilizer (PSS) of different structures^40^, and Memory-based Improved Gorilla Troops Optimizer (MIGTO) for The parameter extraction of the PV model^41^.

To address the drawbacks of the original GTO and improve its performance, this work proposes a modified version called EGTO. In EGTO, the exploration and exploitation update operators are adjusted to enhance exploration at the beginning of the iteration and convergence to the current optimum at the end. a high and low-velocity ratios strategy is introduced to help escape local optima. To evaluate the efficiency of EGTO, it is compared against TSO, GWO, GBO, ARO, POA, RUN, and original GTO algorithms on 23 benchmark functions with different dimensions and CEC2019 benchmark functions. Furthermore, it is used to 9 challenging real-world constrained engineering problems and compared against seven techniques from the literature. Obtained results demonstrate that EGTO achieves exceptionally well, offering advantages such as low time complexity, fast convergence, high solution precision, generality competence, and strength. The main contributions of this paper can be summarized as follows:

- EGTO, an enhanced optimization algorithm based on the Marine Predator Algorithm (MPA) and Gorilla Troops Optimizer (GTO), is proposed to solve global optimization problems and Engineering Design Problems.
- The proposed algorithm (EGTO) is tested on several optimization problems, including twenty-three classical benchmark functions, the IEEE CEC2019 test suite, and seven engineering design problems, and compared with different recent MAs.
- Experimental results suggest that the EGTO technique has a more reliable performance than other comparison optimization algorithms.

The rest of the article is organized as follow: section "The proposed optimization algorithm" presents a brief overview of the basic GTO and the proposed EGTO techniques. Section "Experimental results and discussion" evaluates the performance of the proposed EGTO algorithm on benchmark functions and analyzes the obtained experimental results. In section "EGTO for engineering design problems", the EGTO technique is used to solve seven real-world engineering design problems. Finally, section "Conclusion" concludes the paper and discusses potential research directions.

## The proposed optimization algorithm

This section presents the process of the enhanced GTO (EGTO) based on MPA algorithm. The proposed EGTO algorithm.

### Artificial gorilla troops optimizer (GTO)

#### Exploration phase

During the exploration phase, three distinct operators were employed to advance the investigation: one involved moving to an unfamiliar location, aiming to delve deeper into the GTO algorithm^31^. The second element, transitioning to other gorillas, enhances the equilibrium between exploration and exploitation. Meanwhile, the third element pertains to the exploration phase, where migrating to a familiar position significantly boosts the GTO algorithm's capacity to explore diverse developmental spaces. These distinct operators can be symbolically represented by the following equation:

1 $$\begin{aligned} GX\left( {t + 1} \right) & = \left\{ {\begin{array}{*{20}l} {\left( {ub - lb} \right) \times r_{1} + lb,} \hfill & {rand < z} \hfill \\ {\left( {r_{2} - C} \right) \times X_{r} \left( t \right) + D \times B,} \hfill & {rand \ge 0.5} \hfill \\ {X\left( i \right) - D \times \left( {D \times \left( {X\left( t \right) - GX_{r} \left( t \right)} \right) + r_{3} \times \left( {X\left( t \right) - GX_{r} \left( t \right)} \right)} \right),} \hfill & { rand < 0.5 } \hfill \\ \end{array} } \right. \\ C & = (\cos (2 \times r_{4} ) + 1) \times \left( {1 - \frac{it}{{Maxit}}} \right) \\ D & = C \times k \\ B & = E \times X\left( t \right) \\ E & = \left[ { - C,C} \right] \\ \end{aligned}$$

where $GX\left(t+1\right)$ denotes the gorilla candidate location in the next iteration.$lb$ and $ub$ denote the lower and upper bounds of the variables, respectively. ${r}_{1}$, $rand$,${r}_{2}$,${r}_{3}$, and ${r}_{4}$ refer to random values ranging from 0 to 1. $z$ is a parameter that has a range from 0 to 1.$X\left(t\right)$ is the current vector of the gorilla position while ${X}_{r}\left(t\right)$ denotes a member of the gorillas randomly selected from the entire gorillas and also $G{X}_{r}\left(t\right)$.$k$ denots a random value ranging from -1 to 1.

#### Exploitation phase

During the exploitation phase, two behaviors or strategies are utilized. The first strategy, known as "Follow the silverback," is employed when a certain condition $C\ge W$ is met. Here, $W$ represents a predefined parameter set before the optimization process. Mathematically, the first strategy can be computed as follows^31^:

2 $$\begin{aligned} GX\left( {t + 1} \right) & = D \times M \times \left( {X\left( t \right) - X_{silverback} } \right) + X\left( t \right) \\ M & = \left( {\left| {\frac{1}{N}\mathop \sum \limits_{i = 1}^{N} GX_{i} \left( t \right)} \right|^{g} } \right)^{\frac{1}{g}} \\ g & = 2^{D} \\ \end{aligned}$$

where $GX\left(t+1\right)$ represents the updated position of the gorilla in the search space at the next iteration, ${X}_{silverback}$ is the best solution, $N$ denotes the total number of gorillas. By incorporating this strategy into the optimization process, the algorithm aims to exploit promising regions of the search space by leveraging information from the best solution encountered thus far. This approach enhances the algorithm's ability to converge towards optimal solutions, particularly during the exploitation phase of the optimization process. Overall, Eq. (2) plays a crucial role in guiding the exploration and exploitation of the search space, thereby influencing the algorithm's performance in finding optimal solutions.

The second mechanism is the Competition for adult females and it is used when $C<W$. This mechanism is calculated as follows:

3 $$\begin{aligned} GX\left( i \right) & = X_{silverback} - \left( {X_{silverback} \times Q - X\left( t \right) \times Q} \right) \times A \\ Q & = 2 \times r_{5} - 1 \\ A & = \beta \times H \\ H & = \left\{ {\begin{array}{*{20}c} {N_{1} , rand \ge 0.5} \\ {N_{2} , rand < 0.5} \\ \end{array} } \right. \\ \end{aligned}$$

where $\beta$ refers to a parameter to be given value before the optimization operation.${r}_{5}$ denotes a random value ranging from 0 to 1.

### Proposed EGTO algorithm

The enhanced is called the high and low-velocity ratios based on the MPA algorithm^42^. This way was planned to solve the possibility of the optimum value may drop into local optima. Several recent techniques have been developed based on this strategy and they have been applied for several optimization problems including the parameters of the controller combine proportional-integral-derivative (PID) and fractional order control methods using Enhanced Runge Kutta Optimizer^43^ and optimal size and location of several FACTS devices to achieve minimizing fuel costs and minimizing power losses using Enhanced Tuna Swarm Optimization^44^. This improvement depends on two stages. The first stage is the high-velocity ratio situation. The mathematical model of this stage is as follows:

4.1 $$it<\frac{1}{3}Maxit$$

4.2 $$S = \overrightarrow {{R_{B} }} \otimes \left( {E - \overrightarrow {{R_{B} }} \otimes X_{i} \left( t \right)} \right)$$

4.3 $$X_{i} \left( {t + 1} \right) = X_{i} \left( t \right){ } + P \cdot \overrightarrow {{R_{B} }} \otimes { }S$$

where $\overrightarrow{{R}_{B}}$ denotes a vector of random integers from the Normal distribution that reflect Brownian motion. The notation $\otimes$ refers to entry-by-entry multiplications. The new location is simulated by multiplying $\overrightarrow{{R}_{B}}$ by the previous position, $P$ = 0.5 represents a constant, and $\overrightarrow{{R}_{B}}$ represents a vector of uniform random values in the range [0,1]. This condition happens during the first third of iterations when the step size is large, representative a high level of exploratory ability. The fittest solution (E) is designated as the best location to form a matrix by the following equation^42^:

5 $$E=\left[\begin{array}{ccc}{Xb}_{1.1}^{t}& \cdots & {Xb}_{1.d}^{t}\\ \vdots & \ddots & \vdots \\ {Xb}_{n.1}^{t}& \cdots & {Xb}_{n.d}^{t}\end{array}\right]$$

where $Xb$ is the best solution, which is copied n times to create the E matrix. n is the number of search agents, whereas d denotes the number of dimensions.

The second stage is the low-velocity ratio. This stage happens near the end of the optimization procedure, which is typically related to high exploitation capability. Lévy is the best approach for low-velocity ratios. This stage is represented as:

6.1 $$it>\frac{1}{3}Maxit$$

6.2 $$S = \overrightarrow {{R_{L} }} \otimes \left( {\overrightarrow {{R_{L} }} \otimes E - X_{i} \left( t \right)} \right)$$

6.3 $$X_{i} \left( {t + 1} \right) = E{ } + P \cdot CF \otimes { }S$$

**Figure 2:**
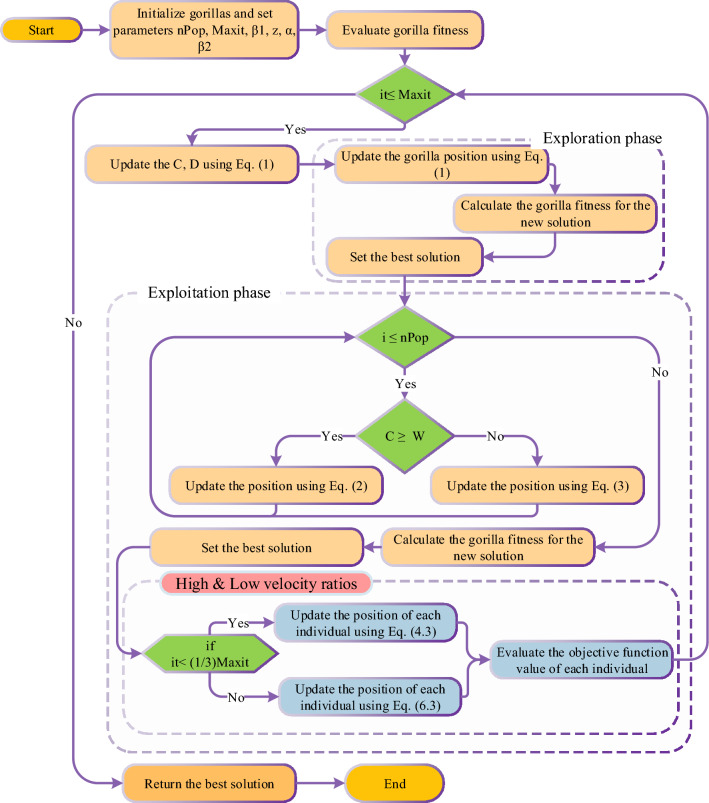
The flowchart of the proposed EGTO technique.

In the Lévy process, multiplying ${R}_{L}$ and E, whereas adding the step size to location assistances in the updating of position. An extra feature of EGTO is increasing the chances of escape from local minima. The flowchart of the proposed EGTO technique is shown in Fig. 2. The place of high and low-velocity ratios in the EGTO technique are displayed in this figure. This modification leads to enhancing the exploration of the EGTO technique. Furthermore, Algorithm 1 defines the EGTO technique’s pseudocode.

**Algorithm 1:**
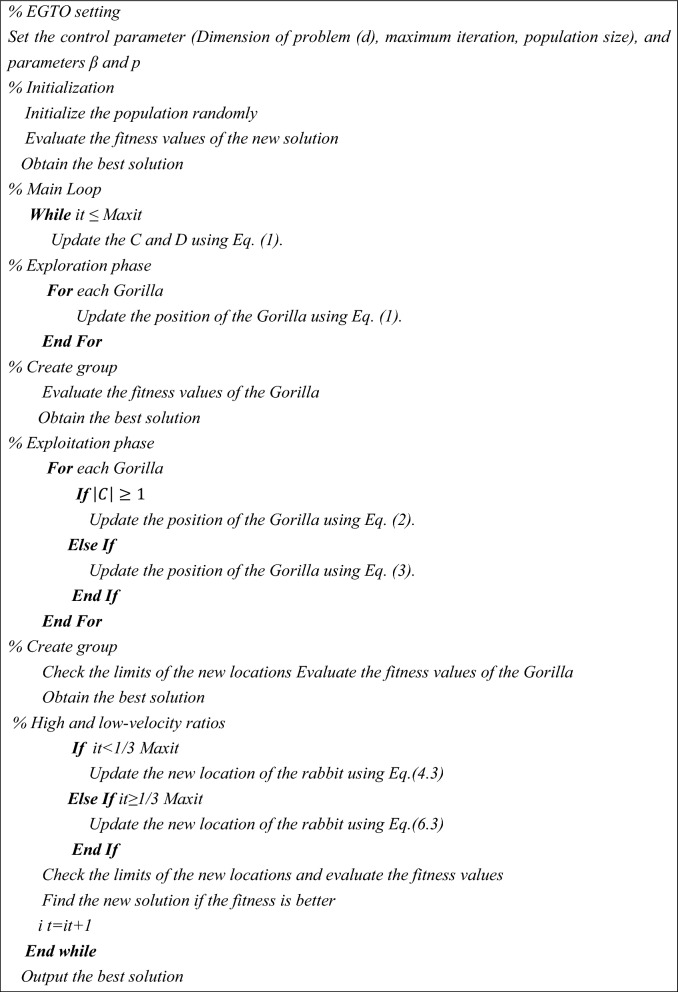
Pseudocode of the EGTO algorithm.

### Computational complexity analysis of the proposed EGTO algorithm

Computational complexity serves as a valuable tool to evaluate the efficiency of algorithms in addressing optimization problems. Several factors influence the complexity of an algorithm, such as the number of individuals engaged (*n*), the dimensionality of the problem's variables (d), and the maximum number of iterations (*Maxit*). For EGTO, the overall computational complexity can be expressed as follows:

$${\text{O}}({\text{EGTO}}) =\mathrm{ O}(\mathrm{problem\, definition}) +\mathrm{ O}({\text{initialization}}) +{\text{O}}(\mathrm{function\, evaluation}) +\mathrm{ O}(\mathrm{Position\, updating\, in\, }Exploration\, phase) +{\text{O}}(\mathrm{Position\, updating\, in\, }Exploitation\, phase) +\mathrm{ O}(\mathrm{Position\, updating\, in\, }High\, and\, low-velocity\, ratios) = O\left(1+n+Tn+\frac{1}{2}Tnd+\frac{1}{2}Tnd+Tnd\right)\cong O(2Tnd+Tn+n)$$.

Compared with the basic techniques, the computational complexity of the EGTO algorithm increases to some extent as a consequence of the introduced High and low-velocity ratios. However, these extra time costs can greatly improve the search performance of the algorithm, which is acceptable based on the NFL theorem^6^.

## Experimental results and discussion

In this section, we utilize a collection of well-known benchmark functions to assess the performance of the proposed EGTO method described in this paper. These benchmark functions encompass three categories: unimodal, multimodal, and multimodal functions with fixed dimensions. The set of unimodal functions consists of seven functions, namely F1–F7. Each of these functions possesses only one global optimal solution. Consequently, they are commonly employed to evaluate the algorithm's ability to exploit local solutions effectively. On the other hand, the multimodal functions F8–F23 not only possess a globally optimal solution but also contain multiple locally optimal solutions. Thus, they serve to challenge the technique's capacity for global exploration and avoidance of local optima. The mathematical formulas and characteristics of these benchmark functions are given in Table A in Supplementary Material.

### Performance of the proposed EGTO algorithm

In this subsection, we assess the effectiveness and performance of the proposed EGTO technique on various benchmark functions. The evaluation involves using statistical measurements, such as best values, mean values, worst values, and standard deviation (STD) to analyze the solutions obtained by the EGTO technique, the original GTO algorithm, and seven other recent optimization algorithms, including the Tuna Swarm Optimization (TSO)^45^, Grey Wolf optimizer (GWO) algorithm^46^, Gradient-based optimizer (GBO) algorithm^47^, Artificial rabbits optimization (ARO)^48^, Pelican Optimization Algorithm (POA)^49^, RUN algorithm^50^, and MPA algorithm^42^. The codes of these algorithms implemented in this study have been provided in Supplementary Material. The results attained with the EGTO algorithm is compared with these recent techniques. To ensure a level playing field, all algorithms, including the proposed one, were evaluated under identical conditions. Specifically, each algorithm was executed for the maximum number of iterations set at 500, with a population size of 50 for 20 independent runs. These computations were conducted using MATLAB R2016a on a Windows 8.1, 64-bit system, with a Core i5-4210U CPU running at 2.40 GHz and 8 GB of RAM. By maintaining consistency in these parameters across all evaluations, we aimed to provide a fair basis for comparison, enabling an objective assessment of algorithmic performance. The parameter settings of the optimization algorithms are shown in Table 1.

**Table 1:** Parameter settings of optimization algorithms for comparison and evaluation of the EGTO.

Algorithms	Parameters setting
GWO	θ (= 2 to 0)
GBO	Pr = 0.5, z = [0,1]
ARO	No such parameter
RUN	
TSO	
POA	
MPA	
GTO	$\beta =3, W=0.8, P=0.03$
EGTO	

In this section, we present a comprehensive assessment of the proposed algorithm's exploitation and exploration capabilities using the previously described unimodal, and multimodal benchmark functions. Tables 2 and 3 display the best values, mean values, worst values, and STD results attained by the EGTO algorithm and other competing algorithms for each function F1–F13 in dimension Dim = 30. The comparison of algorithms in these functions is based on their average values. The Tied rank (TR) technique is utilized to rank these functions, where each technique is assigned, a rank based on its average value, with the algorithm having the smallest average value receiving rank 1, and so on. The algorithm with the lowest TR value is considered the most effective when compared to the other techniques^28^.

**Table 2:** The statistical Results of unimodal benchmark functions using the proposed EGTO technique and other well-known algorithms (Dim = 30).

Function		EGTO	GTO	TSO	GWO	GBO	ARO	POA	RUN	MPA
F1	Best	**0**	1.7E−175	2.4E−115	4.47E−12	9.06E−52	1.59E−26	1.18E−45	9.07E−86	1.68E−07
	Mean	**0**	1.1E−160	6.2E−99	3.12E−11	4.1E−46	1.07E−21	1.62E−37	5.02E−77	5.11E−07
	Median	**0**	7.8E−169	3.4E−106	2.46E−11	1.73E−49	4.68E−23	1.86E−41	1.06E−82	3.6E−07
	Worst	**0**	2.3E−159	1.24E−97	8.73E−11	4.79E−45	7.08E−21	3.09E−36	1E−75	1.81E−06
	Std	**0**	5.1E−160	2.76E−98	2.31E−11	1.27E−45	2.18E−21	6.9E−37	2.24E−76	4.1E−07
	Rank	**1**	2	3	8	5	7	6	4	9
F2	Best	**4.4E−211**	5.76E−87	2.49E−57	1.42E−07	2.03E−28	1.34E−14	3.79E−23	1.04E−47	2.28E−05
	Mean	**3.1E−200**	5.7E−78	4.67E−48	2.77E−07	1.95E−24	1.15E−12	3.15E−20	1.87E−43	0.000156
	Median	**9.7E−206**	5.78E−83	4.53E−53	2.66E−07	1.77E−25	1.22E−13	5.04E−21	1.2E−44	0.000135
	Worst	**6.2E−199**	1.13E−76	9.33E−47	4.78E−07	1.95E−23	1.78E−11	1.67E−19	2.3E−42	0.00043
	Std	**0**	2.53E−77	2.09E−47	9.9E−08	4.64E−24	3.94E−12	4.9E−20	5.35E−43	9.51E−05
	Rank	**1**	2	3	8	5	7	6	4	9
F3	Best	**0**	5E−166	2.3E−106	0.008462	3.42E−42	4.28E−21	1.74E−43	5.75E−77	0.274034
	Mean	**0**	1.7E−144	1.11E−90	0.610441	2.56E−38	5.08E−15	8.76E−39	2.33E−62	3.940563
	Median	**0**	3.2E−156	3.67E−97	0.185412	5.51E−40	6.99E−17	3.91E−41	1.5E−69	3.263723
	Worst	**0**	2.9E−143	2.2E−89	3.567009	2.05E−37	6.41E−14	1.19E−37	4.54E−61	8.369983
	Std	**0**	6.5E−144	4.91E−90	0.827115	5.68E−38	1.51E−14	2.76E−38	1.01E−61	2.31658
	Rank	**1**	2	3	8	6	7	5	4	9
F4	Best	**2.3E−203**	8.81E−88	2.88E−59	0.002608	1.54E−24	8.35E−13	2.06E−24	1.35E−42	0.001671
	Mean	**7.4E−193**	6.68E−78	1.93E−49	0.008	8.59E−22	2.6E−09	5.73E−20	2.09E−35	0.003476
	Median	**2.1E−198**	4.8E−82	1.14E−50	0.007092	1.22E−22	7.79E−10	4.04E−21	5.61E−39	0.003057
	Worst	**9E−192**	9.11E−77	1.66E−48	0.016667	1.08E−20	2.28E−08	4.68E−19	4.16E−34	0.006615
	Std	**0**	2.2E−77	4.07E−49	0.003845	2.47E−21	5.09E−09	1.29E−19	9.3E−35	0.001289
	Rank	**1**	2	3	9	5	7	6	4	8
F5	Best	**4.29E−07**	2.65E−06	0.001195	25.92515	24.25387	0.048127	27.59768	23.34961	25.94347
	Mean	9.225693	2.582855	**0.223669**	27.18903	25.13063	2.57084	28.57786	24.73586	26.61776
	Median	**0.0009**	0.00084	0.052472	27.09814	25.117	1.069097	28.75691	24.85999	26.70742
	Worst	24.69577	25.7961	**2.507327**	28.79035	26.10917	16.26736	28.8694	26.52757	27.5016
	Std	11.60128	7.929365	0.563388	0.72182	0.565603	3.783419	**0.376116**	0.832917	0.397519
	Rank	4	3	**1**	8	6	2	9	5	7
F6	Best	1.06E−08	5.79E−08	7.15E−06	0.252254	0.00018	0.009568	2.287489	**7.23E−09**	0.011749
	Mean	6.64E−08	9.98E−05	0.004004	0.647554	0.00108	0.044563	3.220579	**1.19E−08**	0.068693
	Median	6.8E−08	7.3E−05	0.002184	0.611378	0.000875	0.039666	3.076281	**1.19E−08**	0.063854
	Worst	1.33E−07	0.000315	0.03263	1.172757	0.002836	0.098375	4.6641	**2.24E−08**	0.15925
	Std	3.34E−08	0.000101	0.007109	0.280888	0.000702	0.026373	0.548631	**3.7E−09**	0.037548
	Rank	2	3	5	8	4	6	9	**1**	7
F7	Best	**2.72E−06**	1.95E−05	0.00013	0.001477	0.000111	3.22E−05	1.74E−05	3.74E−05	0.000655
	Mean	0.000114	**0.000113**	0.000598	0.004433	0.001192	0.001407	0.000404	0.00042	0.002961
	Median	0.000102	**8.65E−05**	0.000531	0.003685	0.000985	0.00115	0.000328	0.000413	0.00246
	Worst	0.000293	**0.00029**	0.001638	0.01033	0.004017	0.003564	0.001649	0.000865	0.006116
	Std	8.87E−05	**7.49E−05**	0.000417	0.002554	0.001008	0.001071	0.000361	0.000268	0.001753
	Rank	2	**1**	5	9	6	7	3	4	8
Average Rank		**1.571429**	1.857143	2.857143	7.142857	4.571429	5.142857	5.428571	3.142857	6.857143
Final ranking		**1**	2	3	9	5	6	7	4	8

Bold values have the best performance.

**Table 3:** The statistical Results of multimodal benchmark functions using the proposed technique and other well-known algorithms (Dim = 30).

Function		EGTO	GTO	TSO	GWO	GBO	ARO	POA	RUN	MPA
F8	Best	**−12,569.5**	−12,569.5	−12,569.5	−1495.31	−1872.79	−9902.5	−8611.41	−1660.17	−9098.32
	Mean	**−12,569.5**	−12,569.5	−12,569.4	−1245.57	−1743.83	−9268.23	−7526.88	−1580.75	−8329.76
	Median	**−12,569.5**	−12,569.5	−12,569.4	−1224.18	−1732.31	−9276.94	−7483.79	−1605.45	−8346.16
	Worst	**−12,569.5**	−12,569.2	−12,569	−1123.85	−1659.76	−7798.04	−6352.41	−1397.78	−7695.47
	Std	**0.000478**	0.058006	0.119394	104.0153	58.4135	494.4779	653.4455	78.84949	401.4317
	Rank	**1**	2	3	9	7	4	6	8	5
F9	Best	**0**	**0**	**0**	1.062467	**0**	**0**	**0**	**0**	4.32E−07
	Mean	**0**	**0**	**0**	9.801018	**0**	**0**	**0**	**0**	0.000143
	Median	**0**	**0**	**0**	9.824713	**0**	**0**	**0**	**0**	2.99E−05
	Worst	**0**	**0**	**0**	24.96968	**0**	**0**	**0**	**0**	0.001393
	Std	**0**	**0**	**0**	5.565812	**0**	**0**	**0**	**0**	0.000307
	Rank	**1**	**1**	**1**	9	**1**	**1**	**1**	**1**	8
F10	Best	**8.88E−16**	**8.88E−16**	**8.88E−16**	20.76487	**8.88E−16**	3.29E−14	8.88E−16	**8.88E−16**	4.93E−05
	Mean	**8.88E−16**	**8.88E−16**	**8.88E−16**	20.92344	**8.88E−16**	5.19E−12	2.84E−15	**8.88E−16**	0.000141
	Median	**8.88E−16**	**8.88E−16**	**8.88E−16**	20.94465	**8.88E−16**	1.26E−12	4.44E−15	**8.88E−16**	0.000119
	Worst	**8.88E−16**	**8.88E−16**	**8.88E−16**	21.06309	**8.88E−16**	5.25E−11	4.44E−15	**8.88E−16**	0.000373
	Std	**0**	**0**	**0**	0.083433	**0**	1.17E−11	1.81E−15	**0**	7E−05
	Rank	**1**	**1**	**1**	9	**1**	7	6	**1**	8
F11	Best	**0**	**0**	**0**	6.56E−13	**0**	**0**	**0**	**0**	2.56E−07
	Mean	**0**	**0**	**0**	0.009891	**0**	**0**	**0**	**0**	1.83E−06
	Median	**0**	**0**	**0**	4.55E−12	**0**	**0**	**0**	**0**	1.37E−06
	Worst	**0**	**0**	**0**	0.055407	**0**	**0**	**0**	**0**	5.92E−06
	Std	**0**	**0**	**0**	0.015766	**0**	**0**	**0**	**0**	1.56E−06
	Rank	**1**	**1**	**1**	9	**1**	**1**	**1**	**1**	8
F12	Best	**1.27E−09**	1.45E−09	7.19E−08	0.006066	5.1E−06	0.000211	0.098769	2.99E−09	0.000793
	Mean	**5.78E−09**	3.31E−06	6.45E−05	0.026151	3.6E−05	0.002555	0.203213	6.93E−09	0.00381
	Median	**4.92E−09**	7.71E−07	6.3E−06	0.023474	2.19E−05	0.002594	0.205077	5.58E−09	0.001677
	Worst	1.65E−08	1.97E−05	0.000582	0.047176	0.000217	0.004551	0.359266	**1.57E−08**	0.01777
	Std	3.67E−09	5.82E−06	0.000143	0.013414	4.68E−05	0.001218	0.072077	**3.43E−09**	0.004928
	Rank	**1**	3	5	8	4	6	9	2	7
F13	Best	7.96E−09	**3.35E−09**	1.84E−06	0.09955	0.000369	0.005253	1.610985	4.07E−08	0.032728
	Mean	**7.66E−08**	0.002285	0.003481	0.613832	0.014006	0.038605	2.636569	0.00805	0.09402
	Median	**6.56E−08**	2.96E−05	0.000418	0.609981	0.008366	0.020631	2.980898	0.010987	0.08078
	Worst	**2.26E−07**	0.044065	0.021398	1.044	0.052319	0.218513	2.985918	0.021025	0.336438
	Std	**5.37E−08**	0.009836	0.007162	0.280029	0.016889	0.051532	0.482461	0.008325	0.072531
	Rank	**1**	2	3	8	5	6	9	4	7
Average Rank		**1**	1.666667	2.333333	8.666667	3.166667	4.166667	5.333333	2.833333	7.166667
Final ranking		**1**	2	3	9	5	6	7	4	8

Bold values have the best performance.

Upon examining Table 2, it is evident that the EGTO algorithm outperforms its peers on the majority of the test problems. Specifically, for the unimodal functions (F1–F7), EGTO effectively searches for the global optimum (0) on F1–F4. These results confirm the competitive local exploitation potential of EGTO, particularly for unimodal functions. Moving on to Table 3 (the multimodal functions (F8–F13)), The statistical results of the tied rank on 13 benchmark functions are summarized in these tables. For the multimodal functions, the performance of the proposed algorithm significantly outperforms TSO, GWO, GBO, ARO, RUN, POA, MPA and original GTO. Multimodal functions serve to assess the exploration ability, and thus these results demonstrate the excellent global exploration capability of EGTO. The effectiveness of EGTO in global exploration can be attributed to the designed high and low-velocity ratios, which efficiently expands the unknown search region, enabling the algorithm to bypass local optima and find higher-quality solutions.

In conclusion, the comprehensive assessment reveals that the proposed EGTO algorithm excels in both exploitation and exploration capabilities across various benchmark functions. It outperforms other algorithms on most test problems, indicating its efficacy in finding global optima for unimodal and multimodal functions. The innovative high and low-velocity ratios play a crucial role in enhancing exploration and helps the algorithm in efficiently navigating through the solution space to find high-quality solutions.

**Figure 3:**
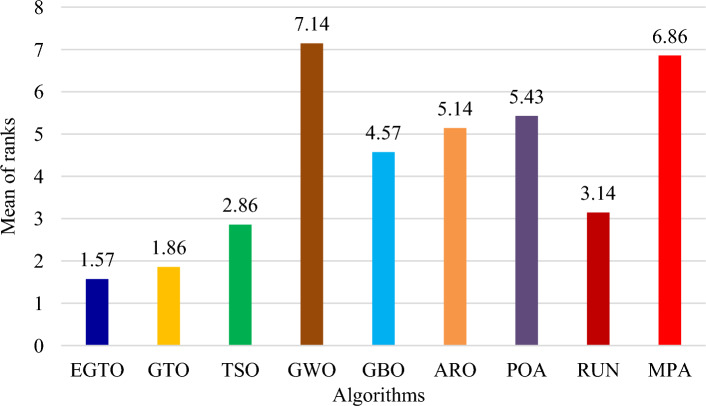
Mean ranks achieved using tied rank test for unimodal functions using several techniques (Dim = 30).

**Figure 4:**
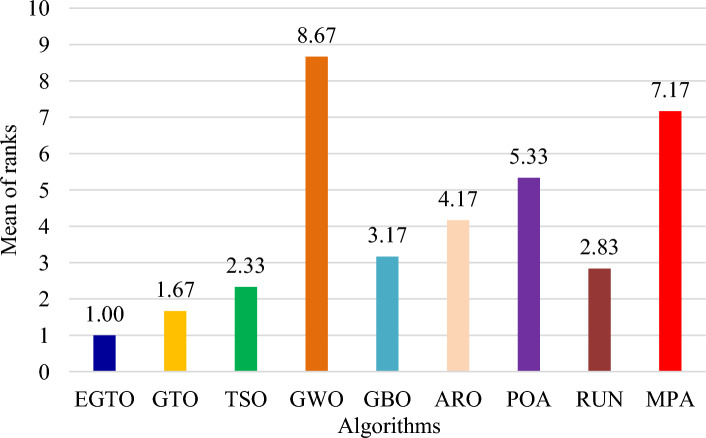
Mean ranks achieved using tied rank test for multimodal functions using several techniques (Dim = 30).

**Figure 5:**
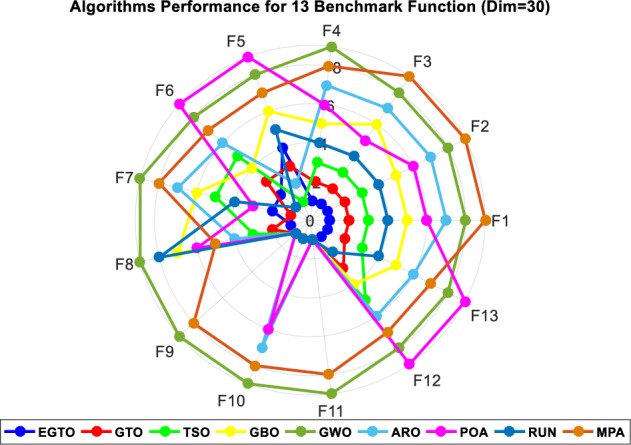
Radar chart for ranks among all compared techniques.

Additionally, the average ranks for all algorithms are presented in Figs. 3 and 4. It is evident from these Figures that the proposed EGTO obtains the lowermost average rank value, indicating that it ranks first among all techniques. Thus, the EGTO technique emerges as the top-performing optimizer in this comparison, according to the tied rank approach. To visualize the ranking of all compared techniques for each function, a radar chart (Fig. 5) is used. This outcome further validates the efficiency of our algorithm in discovering global optima for various problems.

**Figure 6:**
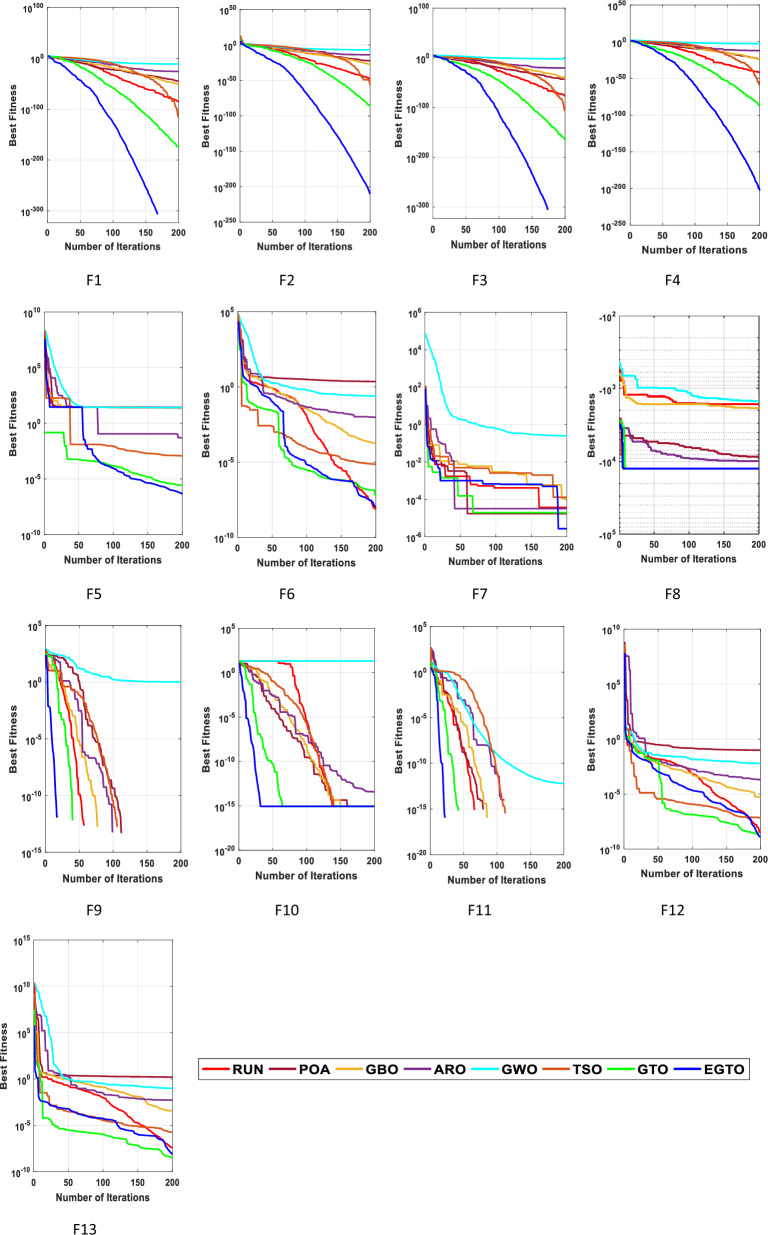
The convergence curves of all algorithms for benchmark functions (Dim = 30).

In the early iterations, search agents in optimization algorithms often undergo dramatic changes to explore promising areas in the search space extensively, and then they gradually converge as the number of iterations increases. To analyze the convergence behavior of the proposed technique in the quest for optimal solutions, Fig. 6 illustrates the convergence curves of TSO, GWO, GBO, ARO, RUN, POA, original GTO, and EGTO on 13 benchmark functions throughout the iterations.

From the figure, it is evident that the proposed EGTO exhibits superior and competitive convergence performance compared to other state-of-the-art algorithms. For the unimodal benchmark functions (F1–F7), EGTO rapidly converges to the global optimum during the initial phases of functions F1–F4, displaying the fastest decay rate in its convergence curve. In contrast, other algorithms experience significant lag and are slower in their search process. This phenomenon arises due to the designed high and low-velocity ratios mechanism, which provides better randomness and population diversity during the initial stage, effectively expanding the search range of EGTO. Despite the multimodal benchmark functions (F8–F13) being more challenging due to several local optima, EGTO maintains excellent convergence behavior in these test cases. These convergence behaviors of EGTO on multimodal functions provide strong evidence that the hybrid operation and high and low-velocity ratios mechanism effectively help escape local optima, contributing to the algorithm's overall robustness and performance.

**Figure 7:**
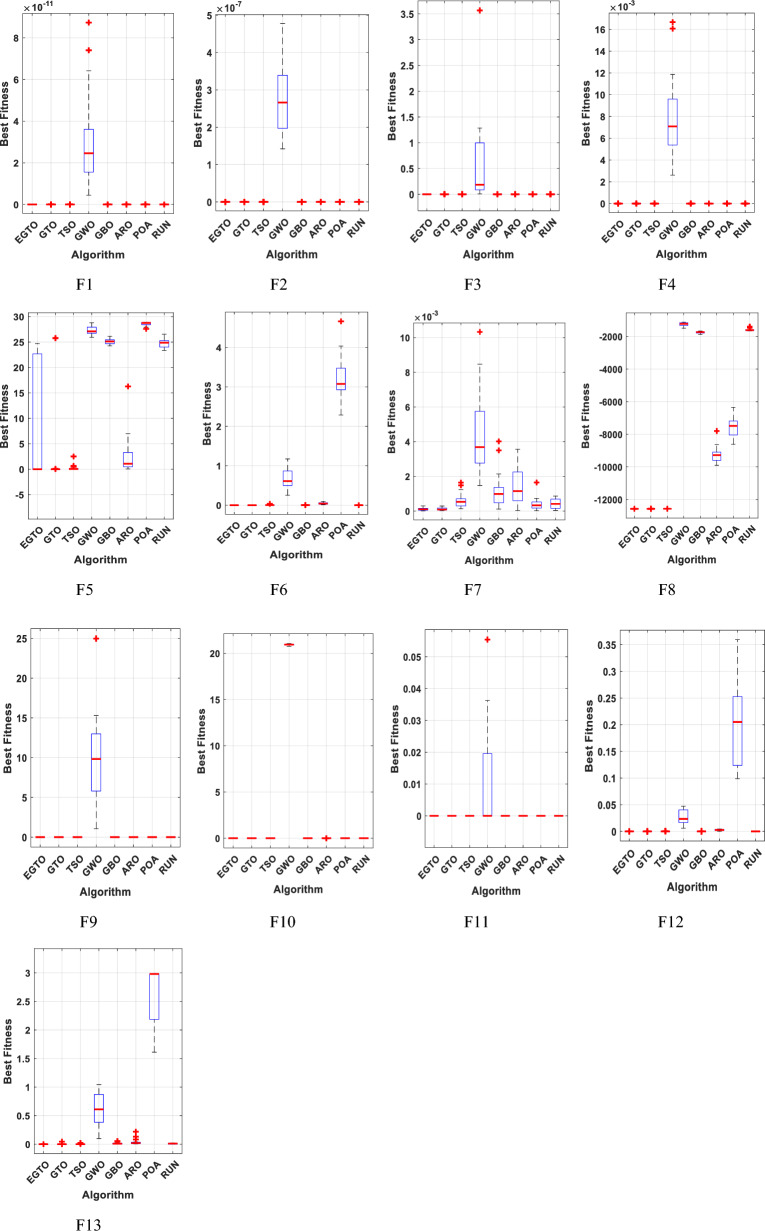
Boxplots for all techniques for benchmark functions (Dim = 30).

The boxplot is a suitable diagram for visualizing data distribution and describing the agreement between the data. In Fig. 7, we present boxplots representing the results obtained from 20 independent runs for 13 representative benchmark functions, comparing EGTO with other algorithms, as shown in Tables 4 and 5. Each box in the figure represents the data distribution for a specific algorithm. The center marker of each box corresponds to the median value, while the bottom and top fringes of the box indicate the first and third quartiles, respectively. Outliers are denoted by the notation "+". By examining Fig. 7, it becomes evident that the proposed EGTO exhibits remarkable consistency and produces no outliers in nearly all test cases during the optimization process.

**Table 4:** The statistical Results of unimodal benchmark functions using the proposed technique and other well-known algorithms (Dim = 50).

Function		EGTO	GTO	TSO	GWO	GBO	ARO	POA	RUN
F1	Best	**0**	3.60E−179	5.50E−109	8.37E−08	3.73E−51	7.67E−27	2.61E−45	4.23E−89
	Mean	**0**	1.10E−157	1.02E−95	2.79E−07	3.59E−45	1.53E−20	3.25E−37	2.9E−67
	Median	**0**	1.00E−167	6.90E−102	2.02E−07	9.57E−47	5.89E−22	1.99E−39	7.55E−78
	Worst	**0**	2.10E−156	1.60E−94	1.11E−06	6.43E−44	1.52E−19	4.39E−36	5.8E−66
	Std	**0**	4.80E−157	3.65E−95	2.39E−07	1.43E−44	3.52E−20	1.01E−36	1.3E−66
	Rank	**1**	2	3	8	5	7	6	4
F2	Best	**8.90E−208**	1.63E−86	1.39E−59	3.69E−05	6.85E−28	9.79E−15	2.54E−23	8.69E−49
	Mean	**3.40E−200**	1.55E−78	2.69E−49	5.89E−05	1.37E−24	1.64E−12	3.58E−19	5.67E−39
	Median	**6.10E−203**	4.74E−81	6.83E−53	5.82E−05	1.86E−25	8.64E−13	4.31E−21	4.76E−43
	Worst	**5.70E−199**	2.92E−77	4.67E−48	8.68E−05	8.53E−24	8.85E−12	2.87E−18	1.13E−37
	Std	**0**	6.52E−78	1.04E−48	1.56E−05	2.62E−24	2.31E−12	8.55E−19	2.52E−38
	Rank	**1**	2	3	8	5	7	6	4
F3	Best	**0**	1.00E−169	2.90E−103	8.247862	3.44E−40	1.46E−19	9.65E−44	3.73E−70
	Mean	**0**	4.10E−149	3.58E−84	69.76243	5.27E−35	9.91E−15	1.83E−36	1.74E−60
	Median	**0**	3.70E−156	6.15E−93	48.49045	7.49E−37	8.75E−16	3.6E−39	3.51E−66
	Worst	**0**	8.10E−148	3.27E−83	261.5955	3.53E−34	7.6E−14	3.23E−35	3.41E−59
	Std	**0**	1.80E−148	9.47E−84	62.21247	9.91E−35	2E−14	7.18E−36	7.63E−60
	Rank	**1**	2	3	8	6	7	5	4
F4	Best	**1.80E−199**	2.78E−89	2.03E−59	0.061477	9.43E−23	1.96E−10	3.48E−23	3.27E−40
	Mean	**7.30E−192**	1.26E−77	1.49E−49	0.208767	1.41E−20	1.58E−08	5.2E−20	3.72E−34
	Median	**8.00E−195**	2.54E−82	1.39E−51	0.199464	1.50E−21	5.61E−09	4.92E−21	7.91E−37
	Worst	**9.00E−191**	2.51E−76	1.49E−48	0.472416	1.03E−19	1.08E−07	5.08E−19	6.37E−33
	Std	**0**	5.61E−77	4.34E−49	0.112809	2.59E−20	2.98E−08	1.28E−19	1.42E−33
	Rank	**1**	2	3	8	5	7	6	4
F5	Best	**3.99E−06**	0.000103	0.00077	46.31971	44.84628	0.09285	48.00589	43.80266
	Mean	10.79961	6.916665	**1.639378**	47.90874	45.75012	1.981324	48.62773	45.11744
	Median	**0.001973**	0.020883	0.134052	47.93871	45.82161	1.318025	48.7604	45.03359
	Worst	44.76108	46.1957	14.40832	48.76858	47.68453	**5.929959**	48.846	47.74144
	Std	19.17784	16.73978	4.366565	0.819451	0.597878	1.772407	**0.261286**	0.950988
	Rank	4	3	**1**	7	6	2	8	5
F6	Best	8.04E−07	9.55E−07	0.00079	2.021396	0.028792	0.065586	6.012652	**9.36E−08**
	Mean	3.17E−06	0.003563	0.069931	2.983071	0.063313	0.196088	7.282118	**1.35E−06**
	Median	3.36E−06	0.001455	0.011559	3.004177	0.060214	0.166124	7.262536	**2.09E−07**
	Worst	5.01E−06	0.012374	0.255212	4.765756	0.165966	0.403829	8.48631	**2.2E−05**
	Std	1.10E−06	0.003987	0.09284	0.650338	0.03347	0.097223	0.786929	**4.86E−06**
	Rank	2	3	5	7	4	6	8	**1**
F7	Best	3.42E−06	1.76E−05	**2.14E−06**	0.001883	7.76E−05	0.000201	3.58E−05	0.000109
	Mean	**0.000108**	0.000148	0.000542	0.007308	0.001522	0.001566	0.000382	0.000632
	Median	**4.94E−05**	0.000158	0.000463	0.006766	0.001334	0.001402	0.000281	0.000452
	Worst	0.000574	**0.000419**	0.001518	0.012483	0.003514	0.003725	0.001003	0.002252
	Std	0.000134	**9.63E−05**	0.000439	0.002513	0.001003	0.001047	0.000274	0.000523
	Rank	**1**	2	4	8	6	7	3	5
Average Rank		**1.57142857**	2.28571429	3.14285714	7.7142857	5.2857143	6.14286	6	3.85714
Final ranking		**1**	2	3	8	5	7	6	4

Bold values have the best performance.

**Table 5:** The statistical Results of multimodal benchmark functions using the proposed technique and other well-known algorithms (Dim = 50).

Function		EGTO	GTO	TSO	GWO	GBO	ARO	POA	RUN
F8	Best	**−20,949.1**	−20,949.1	−20,949.1	−10,764.1	−18,696	−14,037.4	−12,850	-13,481.6
	Mean	**−20,949.1**	−20,949	−20,948.4	−9519.05	−13,997.6	−13,143.8	−10,824.7	-10,484.5
	Median	**−20,949.1**	−20,949.1	−20,949	−9600.68	−13,839.4	−13,134.1	−10,870.6	-10,178.8
	Worst	**−20,949.1**	−20,948.5	−20,942.6	−7944.41	−11,562.5	−12,051.5	−8903.73	-7577.09
	Std	**0.014833**	0.206536	1.572248	728.5785	1579.347	553.937	1025.562	1481.147
	Rank	**1**	2	3	8	4	5	6	7
F9	Best	**0**	**0**	**0**	6.469408	**0**	**0**	**0**	**0**
	Mean	**0**	**0**	**0**	22.52333	**0**	**0**	**0**	**0**
	Median	**0**	**0**	**0**	22.92349	**0**	**0**	**0**	**0**
	Worst	**0**	**0**	**0**	51.82752	**0**	**0**	**0**	**0**
	Std	**0**	**0**	**0**	10.07971	**0**	**0**	**0**	**0**
	Rank	**1**	**1**	**1**	8	**1**	**1**	**1**	**1**
F10	Best	**8.88E−16**	**8.88E−16**	**8.88E−16**	4.96E−05	**8.88E−16**	1.51E−14	**8.88E−16**	**8.88E−16**
	Mean	**8.88E−16**	**8.88E−16**	**8.88E−16**	8.35E−05	**8.88E−16**	3.24E−11	2.49E−15	**8.88E−16**
	Median	**8.88E−16**	**8.88E−16**	**8.88E−16**	8.37E−05	**8.88E−16**	9.76E−12	**8.88E−16**	**8.88E−16**
	Worst	**8.88E−16**	**8.88E−16**	**8.88E−16**	0.000149	**8.88E−16**	2.7E−10	4.44E−15	**8.88E−16**
	Std	**0**	**0**	**0**	2.73E−05	**0**	6.32E−11	1.81E−15	**0**
	Rank	**1**	**1**	**1**	8	**1**	7	6	5
F11	Best	**0**	**0**	**0**	1.19E−07	**0**	**0**	**0**	**0**
	Mean	**0**	**0**	**0**	0.004675	**0**	**0**	**0**	**0**
	Median	**0**	**0**	**0**	3.01E−07	**0**	**0**	**0**	**0**
	Worst	**0**	**0**	**0**	0.035907	**0**	**0**	**0**	**0**
	Std	**0**	**0**	**0**	0.011678	**0**	**0**	**0**	**0**
	Rank	**1**	**1**	**1**	8	**1**	**1**	**1**	**1**
F12	Best	**2.86E−09**	1.24E−06	9.44E−08	0.069288	0.000552	0.001932	0.281402	2.6E−08
	Mean	1.49E−07	7.35E−05	0.000288	0.146249	0.004807	0.008179	0.432569	**5.98E−08**
	Median	1.19E−07	4.01E−05	8.92E−05	0.114348	0.001496	0.006111	0.453748	**6.28E−08**
	Worst	6.24E−07	0.000338	0.001448	0.433898	0.065301	0.018554	0.538911	**9.42E−08**
	Std	1.21E−07	9.59E−05	0.000431	0.096519	0.014259	0.005089	0.070605	**1.92E−08**
	Rank	2	3	4	7	5	6	8	**1**
F13	Best	**2.42E−08**	8.25E−08	2.37E−06	1.561764	0.088822	0.00266	3.969254	1.17E−06
	Mean	**0.000551**	0.000718	0.00413	2.296977	0.580392	0.087569	4.833977	0.011454
	Median	**1.94E−06**	0.000231	0.002076	2.234445	0.253766	0.094081	4.977986	2.37E−05
	Worst	0.010991	**0.002836**	0.030246	3.183965	3.890083	0.177373	4.983704	0.054779
	Std	0.002457	**0.000996**	0.007126	0.411714	1.128273	0.045101	0.353463	0.017196
	Rank	**1**	2	3	7	6	5	8	4
Average Rank		**1.166667**	1.666667	2.166667	7.666667	3	4.166667	5	3.166667
Final ranking		**1**	2	3	8	4	6	7	5

Bold values have the best performance.

Furthermore, the values of the median, maximum, and minimum achieved by EGTO are more tightly concentrated compared to the competitor algorithms. These results highlight the strong stability of the EGTO algorithm proposed in this study. Its ability to consistently perform well across multiple independent runs demonstrates its robustness and reliability. The absence of outliers and the concentration of results indicate that the proposed algorithm consistently converges to high-quality solutions for the benchmark functions analyzed.

Tables 4 and 5 show the statistical results of the EGTO technique and other well-known algorithms when used for unimodal benchmark functions, multimodal benchmark functions, respectively with Dim = 50. The statistical results of the tied rank on 13 benchmark functions are summarized in these tables. After analyzing these Tables, the techniques applied are sorted, and it is observed from the ranking order that the EGTO technique outperforms all the other compared algorithms across 10 benchmark functions problems. The original GTO and TSO also display robust effectiveness as the second and third optimal techniques, respectively.

**Figure 8:**
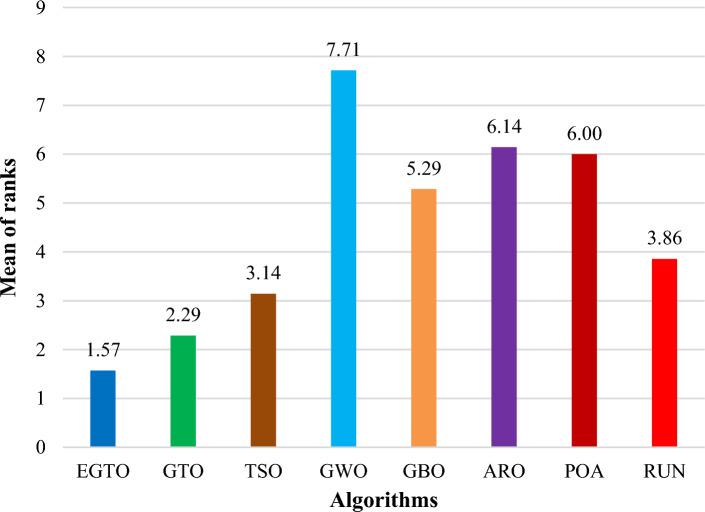
Mean ranks achieved using tied rank test for unimodal functions using several techniques (Dim = 50).

**Figure 9:**
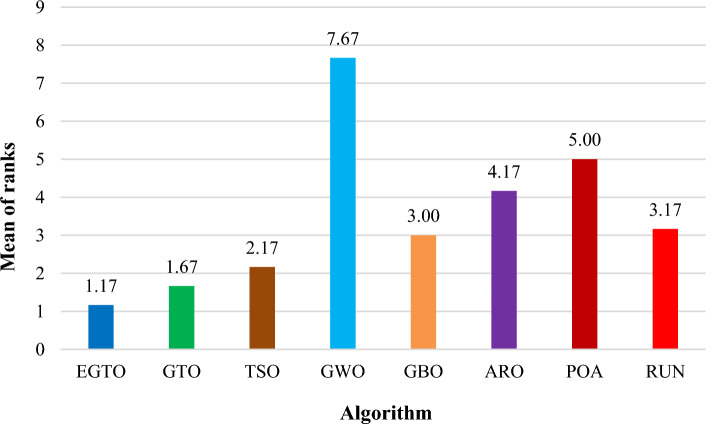
Mean ranks achieved using tied rank test for multimodal functions using several techniques (Dim = 50).

**Figure 10:**
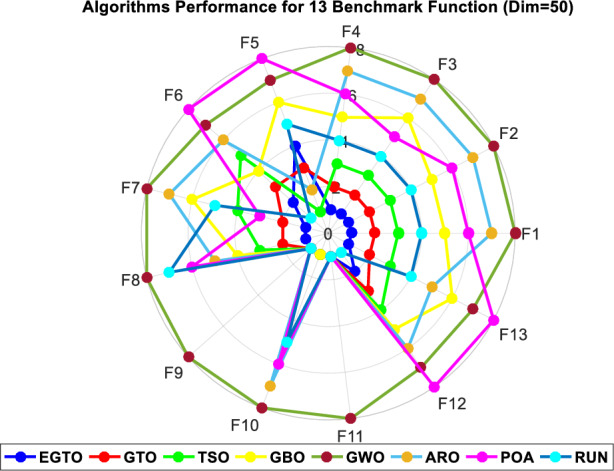
Radar chart for ranks among all compared techniques.

Moreover, the average ranks for all algorithms are displayed in Figs. 8 and 9. It is obvious from these Figures that the EGTO technique achieves the lowermost average rank value, indicating that it ranks first among all techniques. Thus, the EGTO technique emerges as the top-performing algorithm in this comparison, according to the tied rank approach. To visualize the ranking of all compared algorithms for each function, a radar chart (Fig. 10) is used. This outcome further validates the efficiency of our method in discovering global optima for various problems.

**Figure 11:**
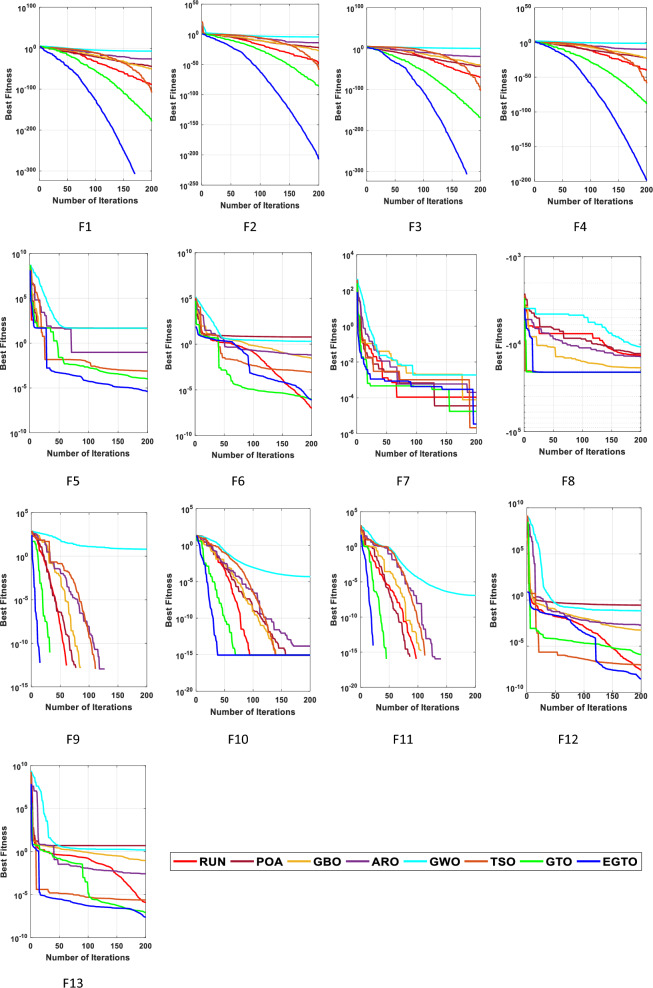
The convergence curves of all algorithms for benchmark functions (Dim = 50).

**Figure 12:**
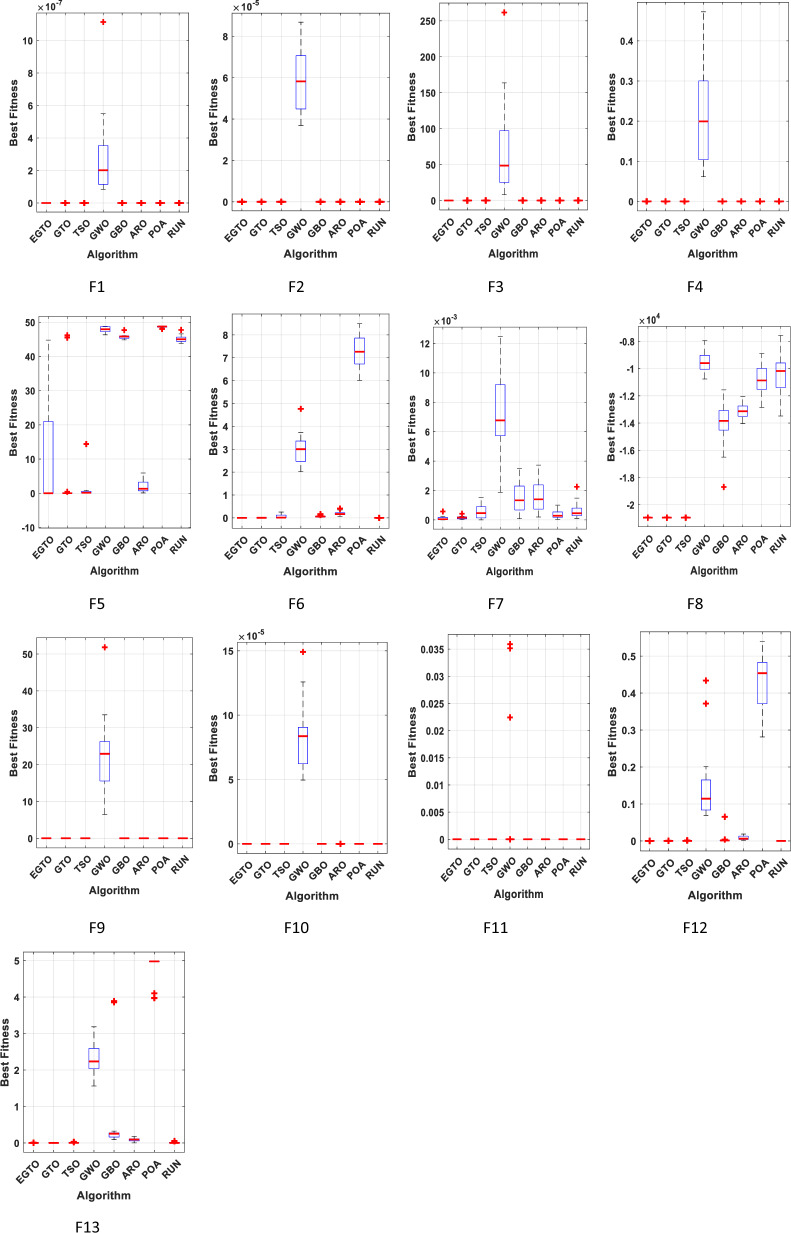
Boxplots for all techniques for benchmark functions (Dim = 50).

From this analysis, it can be concluded that the EGTO technique proves to be a strong technique for solving these types of problems. Further insights into the convergence curves of these techniques for these functions are shown in Fig. 11. For a more in-depth examination into the performance of the EGTO technique, a boxplot of outcomes for each technique and fitness function is displayed in Fig. 12. The boxplots of the EGTO algorithm for most functions are narrow and among the smallest values, indicating its consistency and effectiveness in solving the optimization problems. In summary, the Tied rank comparison displays that the EGTO technique is the most effective algorithm among all the compared methods. The visualizations and statistical analyses provide strong evidence that EGTO efficiently discovers global optima for various problems, making it a highly effective algorithm for solving these types of optimization problems.

This section evaluates the proposed algorithm’s exploration and development capabilities using standard test functions with Dim = 100. The above eight well-known techniques are compared mainly from numerical analysis, boxplots, and convergence curves. After 20 independent runs of the experiment. The obtained results are recorded in Tables 6 and 7 below. It is seen from these tables that the EGTO technique obtained the theoretical optimum value in the 100 dimensions of F1–F13 and had a very stable effect.

**Table 6:** The statistical Results of unimodal benchmark functions using the proposed technique and other well-known algorithms (Dim = 100).

Function		EGTO	GTO	TSO	GWO	GBO	ARO	POA	RUN
F1	Best	**0**	2.30E−174	8.10E−130	0.000811	1.70E−46	1.29E−21	2.6E−43	7.41E−85
	Mean	**0**	5.90E−158	8.21E−91	0.00183	1.25E−41	6.86E−18	8.19E−36	1.81E−69
	Median	**0**	2.30E−165	2.80E−102	0.001685	1.46E−44	4.01E−20	1.73E−39	3.76E−75
	Worst	**0**	1.20E−156	1.64E−89	0.003033	2.47E−40	1.08E−16	1.57E−34	3.04E−68
	Std	**0**	2.70E−157	3.67E−90	0.00067	5.52E−41	2.43E−17	3.5E−35	6.78E−69
	Rank	**1**	2	3	8	5	7	6	4
F2	Best	**9.70E−207**	2.14E−84	4.21E−58	0.006512	3.69E−26	1.99E−13	3.28E−23	6.51E−44
	Mean	**9.60E−194**	2.43E−78	3.28E−50	0.009448	9.27E−24	2.32E−11	5.78E−19	5.34E−38
	Median	**8.60E−200**	1.71E−80	2.37E−52	0.009074	3.54E−24	3.92E−12	5.68E−20	1.14E−40
	Worst	**1.90E−192**	1.56E−77	4.40E−49	0.014826	4.49E−23	1.41E−10	3.51E−18	8.75E−37
	Std	**0**	4.77E−78	9.91E−50	0.002153	1.31E−23	4.26E−11	1E−18	1.96E−37
	Rank	**1**	2	3	8	5	7	6	4
F3	Best	**0**	7.40E−161	1.80E−112	2018.299	6.02E−40	4.55E−18	7.3E−45	1.38E−68
	Mean	**0**	6.00E−143	4.05E−86	6316.698	3.34E−34	6.7E−12	5.38E−38	8.05E−57
	Median	**0**	1.60E−153	1.49E−92	6649.509	5.74E−35	2.96E−13	1.56E−39	3.03E−60
	Worst	**0**	1.20E−141	7.99E−85	10,822.95	2.49E−33	5.27E−11	3.59E−37	7.45E−56
	Std	**0**	2.60E−142	1.79E−85	2716.735	7.51E−34	1.37E−11	1.1E−37	2.24E−56
	Rank	**1**	2	3	8	6	7	5	4
F4	Best	**2.50E−199**	4.61E−87	4.66E−61	2.293994	5.83E−22	1.66E−09	4.3E−23	1.26E−38
	Mean	**7.70E−189**	8.88E−74	8.47E−48	8.76014	4.78E−20	2.35E−07	5.85E−19	5.15E−34
	Median	**8.40E−193**	2.40E−80	3.45E−51	7.281844	3.33E−20	2.1E−08	3.11E−21	8.66E−36
	Worst	**1.10E−187**	1.77E−72	6.25E−47	19.73786	1.71E−19	3.66E−06	8.17E−18	3.26E−33
	Std	**0**	3.97E−73	1.69E−47	4.548566	4.59E−20	8.1E−07	1.9E−18	1.03E−33
	Rank	**1**	2	3	8	5	7	6	4
F5	Best	0.000584	**0.001934**	0.007391	97.51116	95.64811	0.070793	98.5131	94.14901
	Mean	18.83823	**0.14477**	7.037977	98.43943	96.88415	3.136644	98.7123	96.03423
	Median	0.150628	**0.072748**	0.240793	98.47466	97.05474	1.680481	98.72147	95.62332
	Worst	93.8134	**0.587904**	97.24398	99.1791	98.27265	19.86147	98.83727	98.23851
	Std	38.23449	**0.170624**	21.68625	0.456708	0.950938	4.426942	0.081622	1.380597
	Rank	4	**1**	3	7	6	2	8	5
F6	Best	**5.06E−06**	0.00013	6.14E−06	10.70939	0.964525	0.08001	16.11074	0.00093
	Mean	**0.000493**	0.050404	0.290228	12.00791	1.448592	0.608359	18.8955	0.010501
	Median	**0.000446**	0.027816	0.116402	11.93858	1.488376	0.526452	19.19065	0.009442
	Worst	**0.001413**	0.198387	1.282841	13.38338	2.183251	1.72743	20.45441	0.025116
	Std	**0.000328**	0.05592	0.343581	0.810237	0.313971	0.387052	1.0891	0.006585
	Rank	**1**	3	4	7	6	5	8	2
F7	Best	**2.35E−06**	1.46E−05	0.000123	0.00895	8.15E−05	0.000284	5.94E−05	0.00011
	Mean	0.000116	**9.84E−05**	0.000568	0.020018	0.001734	0.00182	0.000322	0.000694
	Median	8.56E−05	**8.27E−05**	0.000581	0.020111	0.001467	0.00194	0.000233	0.000461
	Worst	0.000387	**0.000316**	0.00137	0.031882	0.00598	0.003555	0.00103	0.002003
	Std	0.000101	**7.45E−05**	0.000404	0.006533	0.001358	0.001006	0.000242	0.000575
	Rank	2	**1**	4	8	6	7	3	5
Average Rank		**1.571429**	1.857143	3.285714	7.714286	5.571429	6	6	4
Final ranking		**1**	2	3	8	5	6	6	4

Bold values have the best performance.

**Table 7:** The statistical Results of multimodal benchmark functions using the proposed technique and other well-known algorithms (Dim = 100).

Function		EGTO	GTO	TSO	GWO	GBO	ARO	POA	RUN
F8	Best	**−41,898.3**	**−41,898.3**	**−41,898.3**	−19,825.8	−30,674.8	−21,010.4	−21,752.6	-25,287
	Mean	**−41,897.9**	−41,896.6	−41,895.4	−15,859.2	−25,172.9	−19,270.6	−17,968.1	-20,452.5
	Median	**−41,898.1**	−41,898.1	−41,896.5	−16,291.2	−24,892.7	−19,063.5	−17,659.7	-19,859.8
	Worst	**−41,896.7**	−41,885.4	−41,883	−5262.14	−19,985.3	−17,646.4	−15,643	-17,133.3
	Std	**0.509267**	3.479965	3.850803	2975.467	2569.541	1058.849	1732.901	2290.997
	Rank	**1**	2	3	8	4	6	7	5
F9	Best	**0**	**0**	**0**	34.40649	**0**	**0**	**0**	**0**
	Mean	**0**	**0**	**0**	65.05702	**0**	**0**	**0**	**0**
	Median	**0**	**0**	**0**	58.07168	**0**	**0**	**0**	**0**
	Worst	**0**	**0**	**0**	202.1651	**0**	**0**	**0**	**0**
	Std	**0**	**0**	**0**	34.78852	**0**	**0**	**0**	**0**
	Rank	**1**	**1**	**1**	8	**1**	**1**	**1**	**1**
F10	Best	**8.88E−16**	**8.88E−16**	**8.88E−16**	0.003076	**8.88E−16**	3.14E−13	**8.88E−16**	**8.88E−16**
	Mean	**8.88E−16**	**8.88E−16**	**8.88E−16**	0.004602	**8.88E−16**	3.2E−11	2.49E−15	**8.88E−16**
	Median	**8.88E−16**	**8.88E−16**	**8.88E−16**	0.004568	**8.88E−16**	7.26E−12	**8.88E−16**	**8.88E−16**
	Worst	**8.88E−16**	**8.88E−16**	**8.88E−16**	0.006088	**8.88E−16**	1.45E−10	4.44E−15	**8.88E−16**
	Std	**0**	**0**	**0**	0.000793	**0**	4.34E−11	1.81E−15	**0**
	Rank	**1**	**1**	**1**	8	**1**	7	6	5
F11	Best	**0**	**0**	**0**	0.00058	**0**	**0**	**0**	**0**
	Mean	**0**	**0**	**0**	0.030069	**0**	**0**	**0**	**0**
	Median	**0**	**0**	**0**	0.001257	**0**	**0**	**0**	**0**
	Worst	**0**	**0**	**0**	0.097505	**0**	**0**	**0**	**0**
	Std	**0**	**0**	**0**	0.040877	**0**	**0**	**0**	**0**
	Rank	**1**	**1**	**1**	8	**1**	**1**	**1**	**1**
F12	Best	5.54E−08	**1.21E−08**	1.40E−07	0.264418	0.008684	0.000983	0.525096	9.21E−06
	Mean	**4.52E−06**	0.000268	0.000175	0.433465	0.012255	0.012604	0.668981	5.46E−05
	Median	**4.97E−06**	4.38E−05	3.04E−05	0.413089	0.011187	0.011209	0.672402	5.18E−05
	Worst	**9.11E−06**	0.002795	0.001141	0.743387	0.020355	0.028714	0.919196	0.000135
	Std	**2.67E−06**	0.000624	0.000305	0.134712	0.003447	0.007048	0.095364	3.02E−05
	Rank	**1**	4	3	7	5	6	8	2
F13	Best	**1.19E−06**	2.31E−05	1.93E−06	7.193393	1.211363	0.075641	9.965044	0.010205
	Mean	**0.001434**	0.012835	0.030972	8.221993	4.739807	0.374309	9.97518	0.059776
	Median	**0.000227**	0.010272	0.005917	8.172842	2.365295	0.363753	9.976083	0.049669
	Worst	**0.012355**	0.047149	0.321801	9.433617	9.272248	0.712204	9.983865	0.154541
	Std	**0.003591**	0.013783	0.072832	0.655238	3.616956	0.216487	0.004472	0.041508
	Rank	**1**	2	3	7	6	5	8	4
Average Rank		**1**	1.833333	2	7.666667	3	4.333333	5.166667	3
Final ranking		**1**	2	3	8	4	6	7	4

Bold values have the best performance.

**Figure 13:**
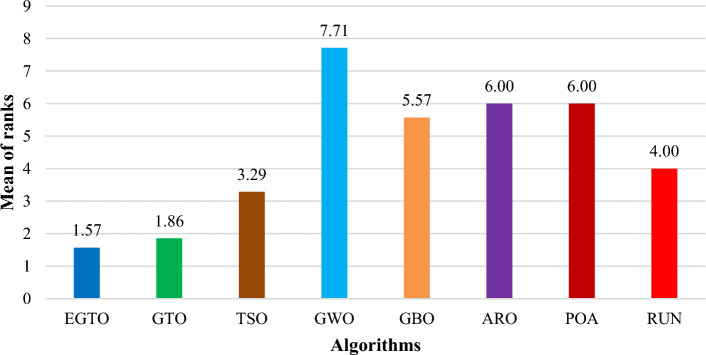
Mean ranks achieved using tied rank test for unimodal functions using several techniques (Dim = 100).

**Figure 14:**
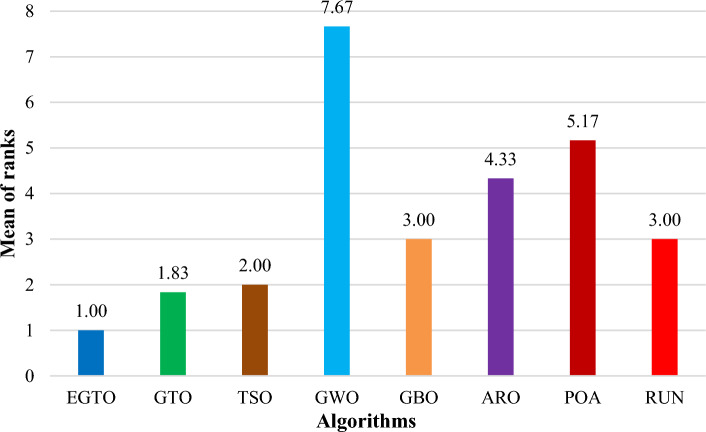
Mean ranks achieved using tied rank test for multimodal functions using several techniques (Dim = 100).

**Figure 15:**
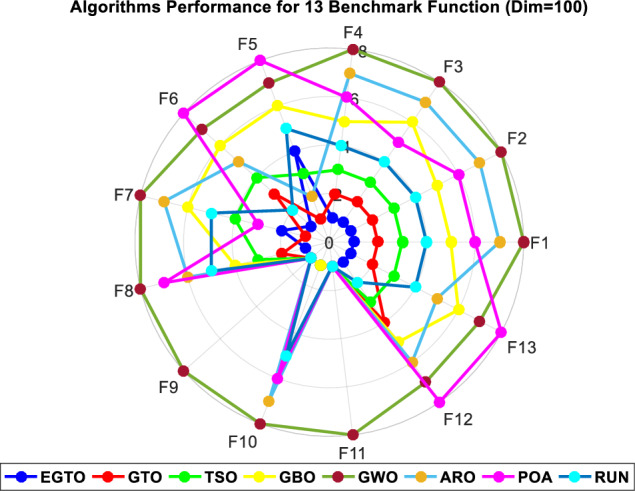
Radar chart for ranks among all compared algorithms.

Additionally, Figs. 13 and 14 present the average ranks for all algorithms. These figures unmistakably demonstrate that the EGTO technique attains the most favorable average rank, signifying its top position among all algorithms. Hence, based on the tied rank approach, the EGTO technique emerges as the best-performing algorithm in this comparison. For a visual representation of the rankings of all compared techniques concerning each function, a radar chart (Fig. 15) has been employed. This outcome provides further validation of the efficiency of our method in discovering global optima for diverse problems.

**Figure 16:**
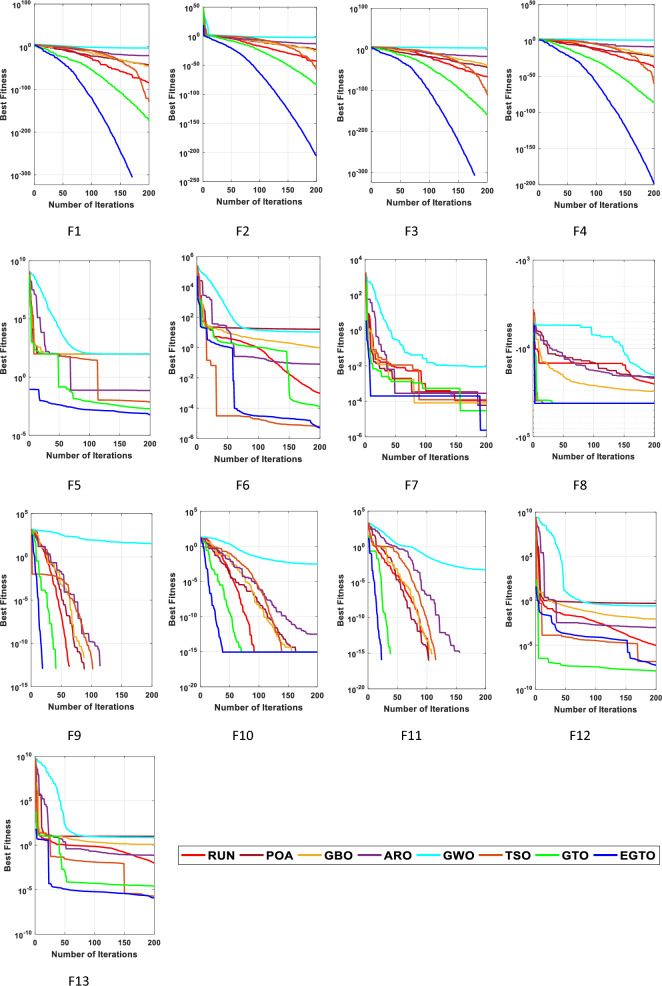
The convergence curves of all algorithms for benchmark functions (Dim = 100).

**Figure 17:**
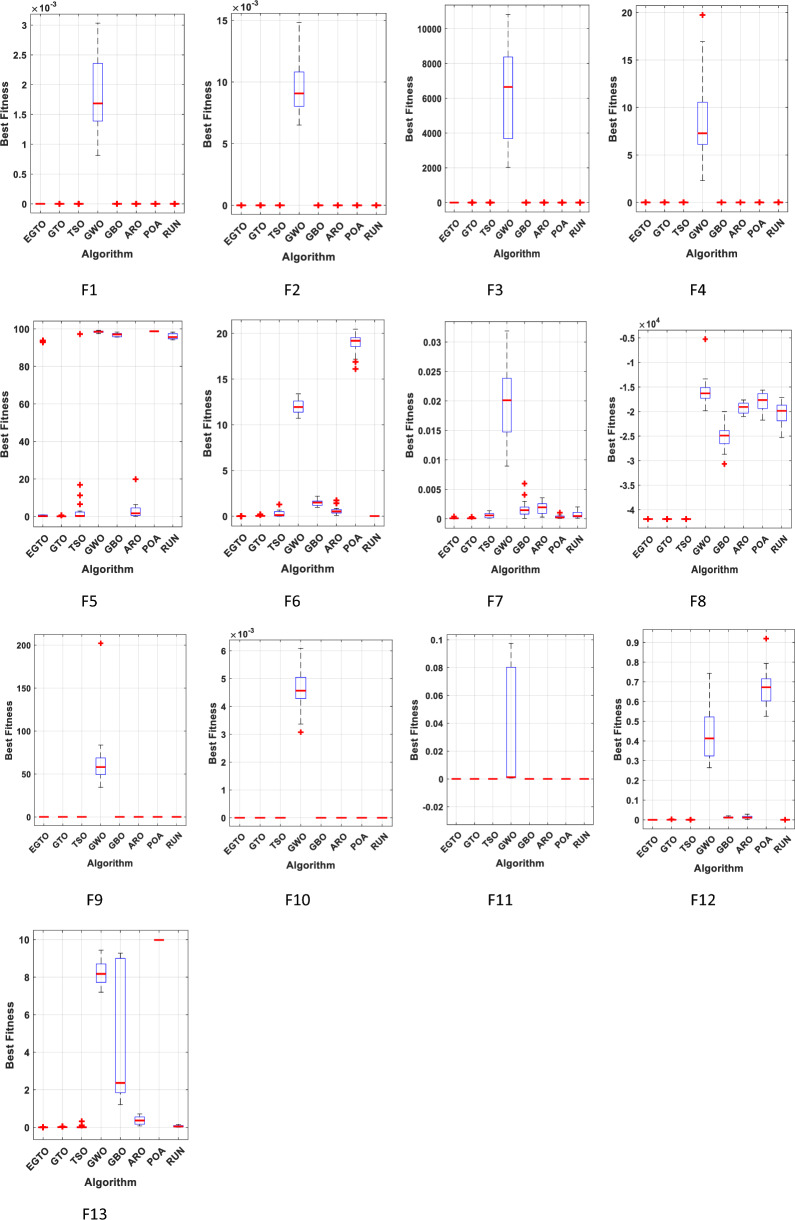
Boxplots for all algorithms for benchmark functions (Dim = 100).

Figure 16 presents the convergence curves of different techniques on 13 illustrative benchmark functions with Dim = 100. From Fig. 16, it is clear that the convergence speed of EGTO is the fastest among all techniques on the most of functions and the EGTO technique can quickly reach the global optimal solution at the beginning of the search process. In order to better demonstrate the constancy of the EGTO technique, the corresponding boxplots of unimodal benchmark functions and multimodal benchmark functions with dimension Dim = 100 are shown in Fig. 17. From Fig. 17, it can be seen that the proposed EGTO technique displays remarkable reliability in most issues with respect to the median, maximum and minimum values compared with the others. Furthermore, the EGTO algorithm generates no outliers during the iterations with the more focused distribution of convergence values, thereby confirming the robust strength and superiority of the proposed EGTO technique. On the basis of experimental results of boxplot analysis and convergence curves, EGTO has a considerable enhancement in convergence speed and permanency compared with the original GTO, which is owed to the good basis of global search placed by high and low-velocity ratios.

Table 8 presents the performance of the EGTO algorithm compared to other well-known algorithms in solving composite benchmark functions. The table provides statistical results for various functions (F14–F23). For each function, the table shows the best, mean, median, and worst values obtained by each algorithm, along with their corresponding standard deviations. Additionally, the table includes the ranks assigned to each algorithm based on their performance for each function. The EGTO algorithm demonstrates excellent performance, achieving the best results (rank 1) for functions F14, F16, F17, F19, F21, F22, and F23. For these functions, EGTO consistently obtains the best solutions, as indicated by the best values being equal to or very close to the optimum. Moreover, EGTO achieves competitive results (rank 2 and 3) for functions F18 and F20, respectively. Overall, the EGTO algorithm exhibits outstanding performance, with an average rank of 1.9, positioning it as the top-performing algorithm among the compared techniques.

**Table 8:** The statistical Results of composite benchmark functions using the proposed technique and other well-known algorithms.

Function		EGTO	GTO	TSO	GWO	GBO	ARO	POA	RUN
F14	Best	**0.998004**	0.998004	0.998004	0.998004	0.998004	0.998004	0.998004	0.998004
	Mean	**0.998004**	0.998004	0.998004	4.76706	1.047705	0.998004	0.998004	2.081493
	Median	**0.998004**	0.998004	0.998004	2.982105	0.998004	0.998004	0.998004	0.998004
	Worst	**0.998004**	0.998004	0.998004	12.67051	1.992031	0.998004	0.998004	10.76318
	Std	**1.02E−16**	1.25E−16	2.04E−16	4.337533	0.222271	2.97E−16	2.94E−13	2.241941
	Rank	**1**	4	4	8	6	2	3	7
F15	Best	**0.000307**	**0.000307**	**0.000307**	0.000329	0.000307	0.000308	0.000307	0.000307
	Mean	0.000359	**0.000307**	0.000335	0.004489	0.001402	0.000441	0.000308	0.00072
	Median	**0.000307**	**0.000307**	0.000314	0.000517	0.000307	0.000404	0.000307	0.000307
	Worst	0.001223	**0.000307**	0.000464	0.020363	0.020363	0.000694	0.000308	0.001223
	Std	0.000205	**1.11E−18**	4.28E−05	0.008145	0.004472	0.000131	5.89E−08	0.000467
	Rank	4	**1**	3	8	7	5	2	6
F16	Best	**−1.03163**	**−1.03163**	**−1.03163**	**−1.03163**	**−1.03163**	**−1.03163**	**−1.03163**	-1.03163
	Mean	**−1.03163**	**−1.03163**	**−1.03163**	**−1.03163**	**−1.03163**	**−1.03163**	**−1.03163**	-1.03163
	Median	**−1.03163**	**−1.03163**	**−1.03163**	**−1.03163**	**−1.03163**	**−1.03163**	**−1.03163**	-1.03163
	Worst	**−1.03163**	**−1.03163**	**−1.03163**	**−1.03163**	**−1.03163**	**−1.03163**	**−1.03163**	-1.03163
	Std	2.04E−16	1.61E−16	**1.02E−16**	7.33E−08	2.04E−16	2.5E−12	1.44E−16	8.13E−12
	Rank	**1**	**1**	**1**	**1**	**1**	7	6	8
F17	Best	**0.397887**	**0.397887**	**0.397887**	0.397887	**0.397887**	0.397887	0.397887	0.397887
	Mean	**0.397887**	**0.397887**	**0.397887**	0.397891	**0.397887**	0.397887	0.397887	0.397887
	Median	**0.397887**	**0.397887**	**0.397887**	0.39789	**0.397887**	0.397887	0.397887	0.397887
	Worst	**0.397887**	**0.397887**	**0.397887**	0.397899	**0.397887**	0.397887	0.397887	0.397887
	Std	**0**	**0**	**0**	3.61E−06	**0**	1.28E−10	0	4.27E−12
	Rank	**1**	**1**	**1**	8	**1**	7	5	6
F18	Best	**3**	**3**	**3**	3.000003	**3**	**3**	**3**	**3**
	Mean	**3**	**3**	**3**	3.000076	**3**	**3**	**3**	**3**
	Median	**3**	**3**	**3**	3.000035	**3**	**3**	**3**	**3**
	Worst	**3**	**3**	**3**	3.000272	**3**	**3**	**3**	**3**
	Std	1.83E−15	1.54E−15	3.82E−15	7.77E−05	1.80E−15	**1.16E−15**	1.53E−15	5.41E−13
	Rank	3	3	3	8	3	**1**	2	7
F19	Best	**−3.86278**	−3.86278	−3.86278	−3.86278	−3.86278	−3.86278	−3.86278	-0.30048
	Mean	**−3.86278**	−3.86278	−3.86278	−3.86113	−3.86278	−3.86278	−3.86278	-0.30048
	Median	**−3.86278**	−3.86278	−3.86278	−3.86243	−3.86278	−3.86278	−3.86278	-0.30048
	Worst	**−3.86278**	−3.86278	−3.86278	−3.8549	−3.86278	−3.86278	−3.86278	-0.30048
	Std	**2.15E−15**	2.22E−15	1.85E−15	0.002706	2.11E−15	3.73E−15	1.49E−10	1.14E−16
	Rank	**1**	4	4	7	4	2	3	8
F20	Best	**−3.322**	**−3.322**	**−3.322**	−3.32198	**−3.322**	**−3.322**	−3.32199	**-3.322**
	Mean	−3.26255	−3.25066	−3.27444	−3.25162	−3.2566	−3.31597	**−3.32197**	-3.28038
	Median	−3.26255	−3.2031	−3.322	−3.31169	−3.2031	−3.322	−3.32197	**-3.322**
	Worst	−3.2031	−3.2031	−3.2031	−3.08936	−3.2031	−3.2031	**−3.32191**	-3.20309
	Std	0.060991	0.059759	0.059759	0.083677	0.060685	0.026567	**2.14E−05**	0.058183
	Rank	5	8	4	7	6	2	**1**	3
F21	Best	**−10.1532**	**−10.1532**	**−10.1532**	−10.1521	−10.1532	−10.1532	−10.1532	-10.1532
	Mean	**−10.1532**	**−10.1532**	**−10.1532**	−9.77157	−8.08129	−10.1187	−10.1489	-8.3689
	Median	**−10.1532**	**−10.1532**	**−10.1532**	−10.1451	−10.1532	−10.1532	−10.1528	-10.1532
	Worst	**−10.1532**	**−10.1532**	**−10.1532**	−2.68162	−4.40109	−9.81141	−10.1062	-5.0552
	Std	**2.61E−15**	**2.61E−15**	4.43E−15	1.668806	2.607259	0.090338	0.01199	2.494761
	Rank	**1**	**1**	**1**	6	8	5	4	7
F22	Best	**−10.4029**	**−10.4029**	**−10.4029**	−10.4028	−10.4029	−10.4029	−10.4029	-10.4029
	Mean	**−10.4029**	**−10.4029**	**−10.4029**	−10.1316	−7.89296	−10.1364	−10.137	-9.07412
	Median	**−10.4029**	**−10.4029**	**−10.4029**	−10.3952	−10.4029	−10.4029	−10.4029	-10.4029
	Worst	**−10.4029**	**−10.4029**	**−10.4029**	−5.11735	−2.7659	−5.07631	−5.08767	-5.08767
	Std	3.51E−15	3.87E−15	**2.41E−15**	1.180232	3.186811	1.191013	1.188484	2.36137
	Rank	**1**	**1**	**1**	6	8	5	4	7
F23	Best	**−10.5364**	**−10.5364**	**−10.5364**	−10.5327	−10.5364	−10.5364	−10.5364	-10.5364
	Mean	**−10.5364**	**−10.5364**	**−10.5364**	−9.71635	−6.97401	−10.1522	−10.5363	-9.18443
	Median	**−10.5364**	**−10.5364**	**−10.5364**	−10.528	−5.12848	−10.5364	−10.5363	-10.5364
	Worst	**−10.5364**	**−10.5364**	**−10.5364**	−2.42132	−2.80663	−3.83543	−10.5357	-5.12848
	Std	3.02E−15	2.70E−15	**1.68E−15**	2.494843	3.064853	1.502943	0.0002	2.402536
	Rank	**1**	**1**	**1**	6	8	5	4	7
Average Rank		**1.9**	2.5	2.3	6.5	5.2	4.652174	4.608696	4.913043
Final ranking		**1**	3	2	8	7	5	4	6

Bold values have the best performance.

**Figure 18:**
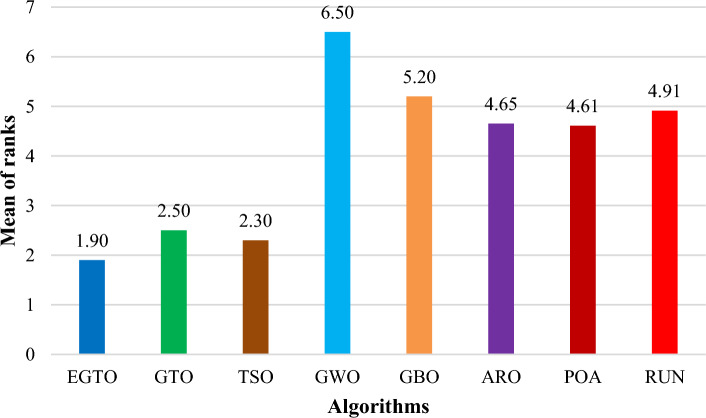
Mean ranks attained using tied rank test for multimodal functions with fixed dimensions using several techniques.

**Figure 19:**
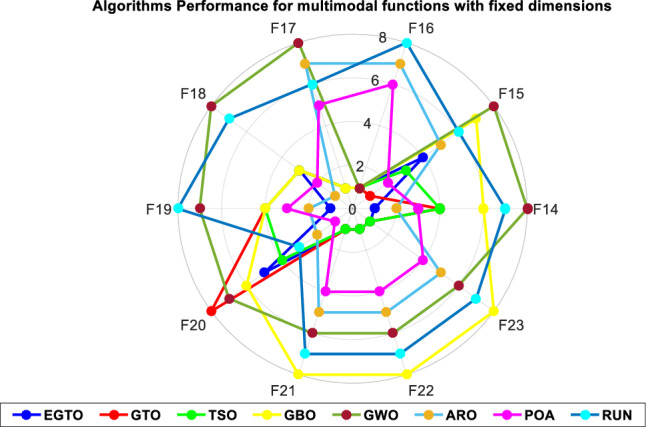
Radar chart for ranks among all compared algorithms.

Figure 18 illustrates the mean ranks attained using the tied rank test for multimodal functions with fixed dimensions using the EGTO technique and several other techniques. The tied rank test is a statistical analysis that allows for a fair and unbiased comparison of algorithms' performance on different benchmark functions. A lower mean rank indicates better performance, as it suggests that the algorithm achieved more favorable results across the various benchmark functions. In the graph, we observe that the EGTO algorithm consistently achieves low mean ranks for most multimodal functions. This indicates that EGTO outperforms other algorithms on a majority of benchmark functions in terms of finding high-quality solutions. The low mean ranks further highlight EGTO's robustness and effectiveness in handling a diverse set of complex multimodal functions. Comparing the mean ranks of EGTO with other algorithms, we can see that EGTO generally outperforms its competitors, demonstrating its superiority in solving multimodal optimization problems with fixed dimensions. The graph's overall trend shows that EGTO consistently ranks higher than other algorithms, reinforcing its reputation as a powerful and reliable optimization technique for challenging multimodal functions. Moreover, Fig. 19 provides compelling evidence of the EGTO algorithm's exceptional performance across various multimodal functions with fixed dimensions, making it a valuable and competitive choice for researchers and practitioners seeking efficient solutions to complex optimization problems.

**Figure 20:**
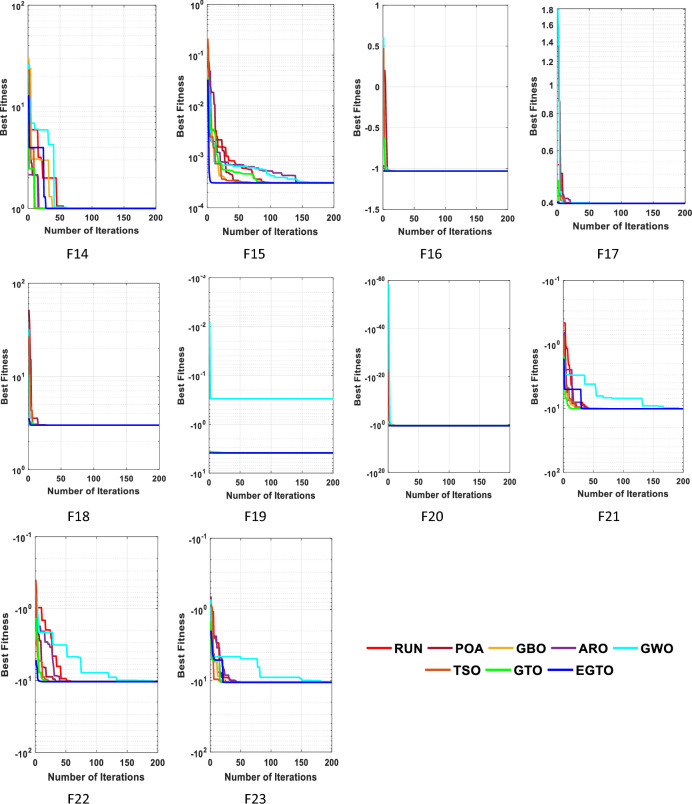
The convergence curves of all techniques for benchmark functions.

Figure 20 displays the convergence curves for each algorithm. Observing these plots, it becomes evident that the EGTO algorithm exhibited robust convergence and exploitation capabilities, efficiently reaching optimal values. Notably, the EGTO algorithm outperformed other methods, demonstrating faster convergence and greater stability. Its superior performance surpasses that of the other algorithms significantly.

**Figure 21:**
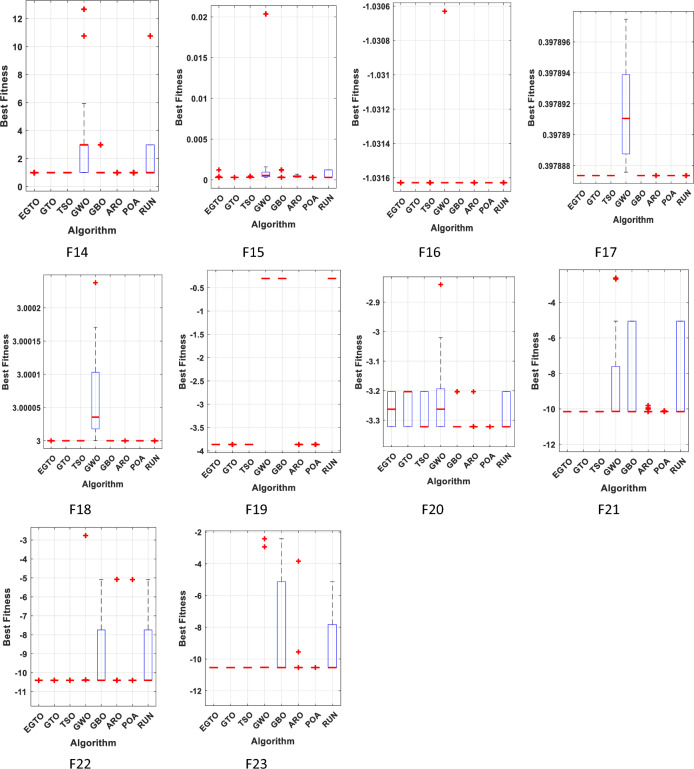
The boxplots of studied techniques for benchmark functions.

Boxplots are drawn in this paper to visualize the distribution of the data. It can well describe the consistency between data. Figure 21 depicts the boxplot of EGTO against seven other comparison techniques over ten representative benchmark functions. As can be seen from this Figure, the EGTO algorithm has better consistency than other original algorithms. No outliers were generated during the iteration. The obtained median, maximum and minimum values are more concentrated than other comparison algorithms. The above confirms the high stability of the EGTO technique.

### The performance of the EGTO technique for the CEC2019 function

The benchmarking of algorithms utilized the CEC2019 function, and their performance evaluation was carried out using the challenging benchmark functions from the CEC2019 test suite, as presented in Table B in Supplementary Material^51^. This test set comprises highly difficult and complex composition functions, providing a rigorous assessment for the algorithms. This test suite encompasses ten intricate and contemporary benchmark functions, whose profiles are detailed in Appendix (C). As mentioned in the preceding subsection, the proposed EGTO and seven other comparison algorithms were independently executed 20 times on each function, with fixed parameters of maximum iteration (300) and population size (50).

The best, mean, median, worst values and standard deviation results obtained from this test are displayed in Table 9. The analysis from Table 9 indicates that EGTO outperforms the other seven algorithms in 5 out of 10 test functions. Furthermore, in comparison to its peers, EGTO achieves the best mean ranking value of 2.3, closely followed by the ARO algorithm. These findings underscore EGTO's capability in tackling a wide range of challenging optimization problems. In summary, this section thoroughly verifies the efficiency and superiority of the EGTO method through a series of experiments on classical benchmark functions and the IEEE CEC2019 test suite. Whether dealing with simple or complex numerical problems, EGTO consistently delivers satisfactory results. It successfully builds upon the strengths of the basic GTO while incorporating the high and low-velocity ratios to address issues related to poor population diversity, falling into local optima, and the balance between exploration and exploitation.

**Table 9:** The statistical Results of CEC 2019 benchmark functions by the EGTO algorithm and other recent techniques.

Function		EGTO	GTO	TSO	GWO	GBO	ARO	POA	RUN
CEC01	Best	**33,618.08**	35,594.33	38,367.19	1,423,853	36,588.1	38,274.25	37,259.11	37,559.33
	Mean	**36,196.98**	37,717.69	41,253.04	1.65E + 08	38,473.22	42,195.47	39,295.65	39,673.84
	Median	**36,249.13**	37,702.91	40,989.24	83,361,980	38,168.5	42,720.13	38,853.25	39,357.95
	Worst	**37,741.19**	38,614.65	44,465.01	8.71E + 08	40,520.11	46,664	45,255.64	42,946.15
	Std	1025.663	**702.0736**	1891.972	2.11E + 08	997.8881	2621.721	1984.379	1494.939
	Rank	**1**	2	6	8	3	7	4	5
CEC02	Best	**18.34286**	18.34286	18.34286	18.34352	18.34286	18.34286	18.34286	18.34286
	Mean	**18.34286**	18.34286	18.34286	18.34413	18.34286	18.34286	18.34306	18.34287
	Median	**18.34286**	18.34286	18.34286	18.34415	18.34286	18.34286	18.34309	18.34286
	Worst	**18.34286**	18.34286	18.34286	18.34474	18.34286	18.34286	18.34357	18.34289
	Std	**3.05E−15**	3.26E−15	8.83E−12	0.000412	4.75E−15	2.2E−07	0.000212	7.04E−06
	Rank	**1**	3	3	8	3	2	7	6
CEC03	Best	**13.7024**	**13.7024**	**13.7024**	**13.7024**	**13.7024**	13.7024	13.7024	13.7024
	Mean	**13.7024**	**13.7024**	**13.7024**	**13.7024**	**13.7024**	13.7024	13.7024	13.7024
	Median	**13.7024**	**13.7024**	**13.7024**	**13.7024**	**13.7024**	13.7024	13.7024	13.7024
	Worst	**13.7024**	**13.7024**	**13.7024**	**13.7024**	**13.7024**	13.7024	13.7024	13.7024
	Std	1.95E−15	**1.82E−15**	2.48E−15	3.72E−08	**1.82E−15**	2.51E−09	5.69E−09	3.31E−12
	Rank	**1**	**1**	**1**	**1**	**1**	7	8	6
CEC04	Best	17.91429	17.91429	40.79822	42.06228	2.989918	**13.77326**	57.46166	30.86298
	Mean	74.04839	70.29808	89.50047	65.03689	37.66404	**28.58871**	564.9421	96.28744
	Median	75.81271	51.24516	73.13411	62.73346	34.82852	**27.49683**	128.3196	94.54379
	Worst	163.1759	184.0677	173.1232	90.53712	72.6365	**51.15236**	2639.438	176.1114
	Std	41.2218	44.65757	38.16052	15.88823	20.56157	**10.77636**	827.6635	35.37281
	Rank	5	4	6	3	2	**1**	8	7
CEC05	Best	2.046796	2.06399	2.044278	2.066691	2.031999	**2.008355**	2.170759	2.05908
	Mean	2.228032	2.273414	2.201004	2.382897	2.145449	**2.086208**	2.447166	2.261986
	Median	2.21787	2.230084	2.151298	2.258665	2.124296	**2.084869**	2.362915	2.224112
	Worst	2.553449	2.586235	2.545979	2.913336	2.376441	**2.229521**	3.239553	2.689202
	Std	0.12872	0.150356	0.128846	0.290806	0.079564	**0.049679**	0.303748	0.175315
	Rank	4	6	3	7	2	**1**	8	5
CEC06	Best	**4.136064**	7.664578	10.32064	9.81309	10.92985	5.950698	8.647794	5.621859
	Mean	**6.998419**	9.585144	11.97105	11.71785	12.15042	7.86019	10.13625	9.539735
	Median	**6.720673**	9.533209	12.06164	11.77171	12.03042	7.884115	10.0589	9.301279
	Worst	10.74191	11.228	12.88728	12.96769	13.47917	**9.334014**	11.30344	12.45787
	Std	1.483926	1.066953	0.693314	0.913684	0.694779	**1.015162**	0.683371	1.986566
	Rank	**1**	4	7	6	8	2	5	3
CEC07	Best	46.53208	22.32578	92.34569	27.78967	1.765186	117.0165	38.44678	**16.15633**
	Mean	**152.7594**	337.7829	438.5803	480.6181	277.2635	195.6447	178.7052	171.6093
	Median	**99.82271**	339.307	327.9422	411.3108	244.1602	170.9214	169.9344	172.8656
	Worst	**400.9307**	802.2705	1089.658	935.489	592.9046	514.1951	521.0228	443.8192
	Std	107.193	194.8108	286.7491	267.7199	170.829	85.08222	112.2538	**86.01406**
	Rank	**1**	6	7	8	5	4	3	2
CEC08	Best	3.444135	3.788167	3.861537	2.955845	3.678619	**1.450394**	2.847841	2.798637
	Mean	4.972668	5.209999	5.676547	4.996896	4.531443	**3.797536**	4.351537	4.70436
	Median	5.159898	5.195812	5.915016	4.885166	4.425442	**3.933821**	4.408483	4.519055
	Worst	6.25621	6.183235	6.781473	6.525674	6.200601	**4.846654**	5.065779	6.319478
	Std	0.726541	0.692929	0.920203	0.994574	0.694315	0.831919	**0.531198**	1.000729
	Rank	5	7	8	6	3	**1**	2	4
CEC09	Best	3.352172	3.371196	3.45287	3.828936	**3.384436**	3.419661	4.262487	3.47651
	Mean	3.505504	3.660784	3.728926	5.530333	**3.488992**	3.658246	5.667908	5.254113
	Median	3.422031	3.551359	3.682639	5.633096	**3.476246**	3.606088	5.480828	5.496025
	Worst	4.134973	4.443227	4.119344	7.418969	**3.758279**	4.476398	7.460838	6.460125
	Std	0.237965	0.310184	0.180103	0.960602	**0.104664**	0.227759	0.852676	0.978177
	Rank	2	4	5	7	**1**	3	8	6
CEC10	Best	3.813597	20.98695	21.27261	21.29007	21.19943	**1.190124**	6.874329	21.10792
	Mean	20.15596	21.10011	21.52002	21.51929	21.45935	**19.99603**	20.47897	21.43253
	Median	**21**	21.06452	21.55142	21.52565	21.45471	21.06202	21.20879	21.44123
	Worst	**21.22352**	21.42933	21.64499	21.64796	21.62638	21.23736	21.36953	21.68665
	Std	3.84692	0.117979	0.107606	**0.100104**	0.104602	4.445576	3.206165	0.125027
	Rank	2	4	8	7	6	**1**	3	5
Average Rank		**2.3**	4.1	5.4	6.1	3.4	2.9	5.6	4.9
Final ranking		**1**	4	6	8	3	2	7	5

Bold values have the best performance.

**Figure 22:**
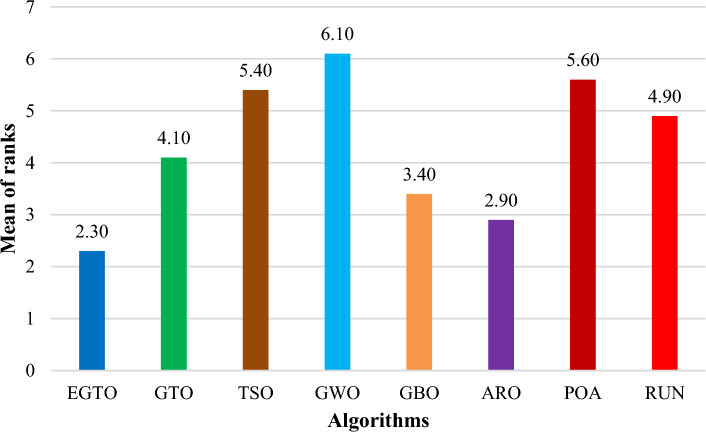
Mean ranks achieved using tied rank test for CEC 2019 benchmark functions using several techniques.

**Figure 23:**
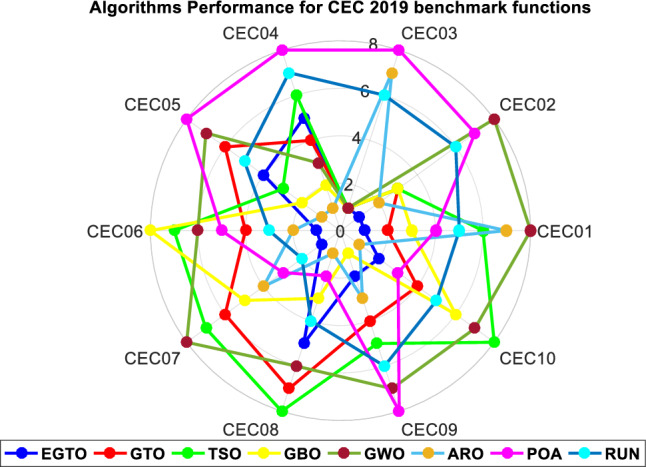
Radar chart for ranks among all studied techniques.

Figure 22 displays the mean ranks obtained by the tied rank test for the CEC 2019 benchmark functions using various algorithms. The graph provides a comprehensive comparison of the performance of different algorithms across these challenging benchmark functions. Mean ranks offer valuable insights into the relative effectiveness of each algorithm, enabling a clearer understanding of their overall performance. On the other hand, Fig. 23 presents a radar chart that visualizes the ranks achieved by all compared algorithms. This chart offers a concise and intuitive representation of how each algorithm ranks in comparison to others across the benchmark functions. The radar chart's multi-dimensional nature allows for a quick assessment of algorithmic strengths and weaknesses in tackling the diverse set of problems present in the CEC 2019 benchmark. These figures unmistakably demonstrate that the EGTO technique attains the most favorable average rank, signifying its top position among all techniques.

**Figure 24:**
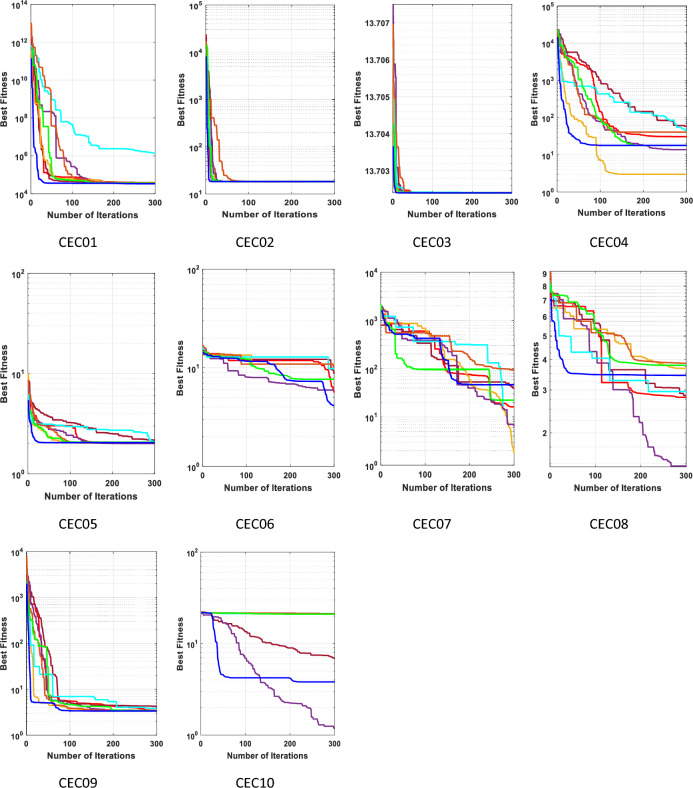
The convergence curves of all algorithms for CEC 2019 benchmark functions.

**Figure 25:**
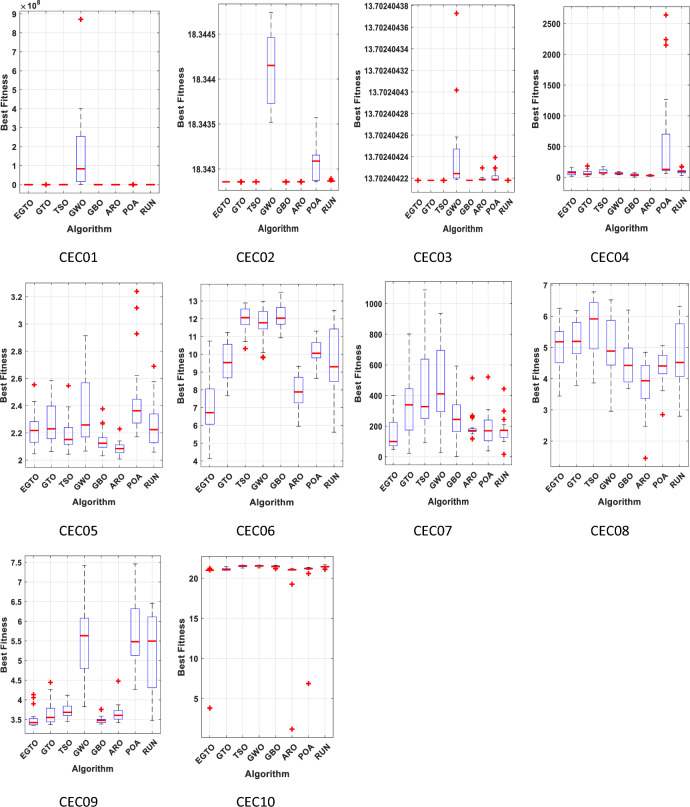
Boxplots for all algorithms for CEC 2019 benchmark functions.

The convergent graph of the implemented algorithms is illustrated in Fig. 24. Observing these curves, it becomes evident that EGTO exhibits comparable convergence for the majority of the functions. Figure 25 presents a box plot depicting a comparison of the algorithms, including the proposed EGTO, for solving the functions. The box plot analysis clearly demonstrates that EGTO outperforms the competitor metaheuristic algorithms, as it showcases a smaller width and a more efficient center, indicating its superior performance. Additionally, the results suggest that EGTO achieves more consistent and robust solutions across the tested functions.

## EGTO for engineering design problems

In this section, we apply the proposed EGTO algorithm to solve a set of classical engineering problems. These problems involve various constraints, both in terms of equalities and inequalities. We compare the performance of the EGTO technique against commonly used optimization techniques when applied to these engineering design problems. The selected problems for analysis are well-known in the field and include the Three-Bar Truss Design (BTD), Compression Spring Design (CSD), Pressure Vessel Design (PVD), Cantilever Beam Design (CBD), Welded Beam Design (WBD), Speed Reducer Design (SRD), and Gear Train Design (GTD). Metaheuristic algorithms have proven to be effective in addressing such constrained engineering problems. To assess the efficacy of the proposed EGTO algorithm, we compare its results with those obtained using other metaheuristic algorithms, including the original GTO, TSO, GWO, GBO, ARO, POA, and RUN algorithms. For consistency, we assume the same simulation assumptions as outlined in the previous section.

### Three-bar truss design problem

**Figure 26:**
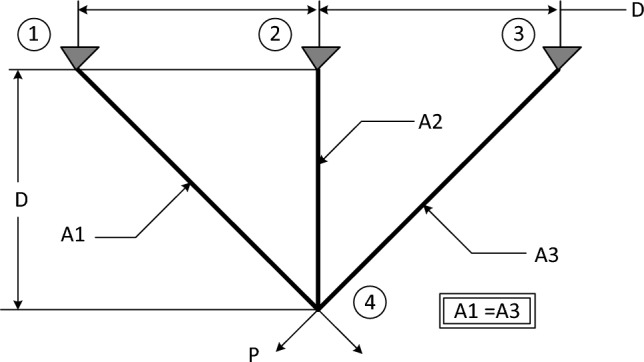
Schematic of three-bar truss problem.

The 3-bar truss design (3BTD) is a prominent problem in the field of civil engineering, involving the manipulation of two parameters to attain the minimum weight when designing a truss^52^. Figure 26 visually represents the layout of this problem. The mathematical model of this design is formulated as follows.

$$\begin{aligned} {\text{Minimize }}\quad f\left( {A1,A2} \right) & = l \times \left( {2\sqrt 2 x_{1} + x_{2} } \right) \\ {\text{Subject}}\;{\text{ to }}\quad G_{1} & = \frac{{\sqrt 2 x_{1} + x_{2} }}{{\sqrt 2 x_{1}^{2} + 2x_{1} x_{2} }}P - \sigma \le 0, \\ G_{2} & = \frac{{x_{2} }}{{\sqrt 2 x_{1}^{2} + 2x_{1} x_{2} }}P - \sigma \le 0 \\ G_{3} & = \frac{1}{{\sqrt 2 x_{2} + x_{1} }}P - \sigma \le 0 \\ \end{aligned}$$

where $l=100 cm;P=\frac{2kN}{c{m}^{2}};\sigma =\frac{2kN}{c{m}^{2}}$; Variable range: $0\le {x}_{1},{x}_{2} \le 1.00$.

**Figure 27:**
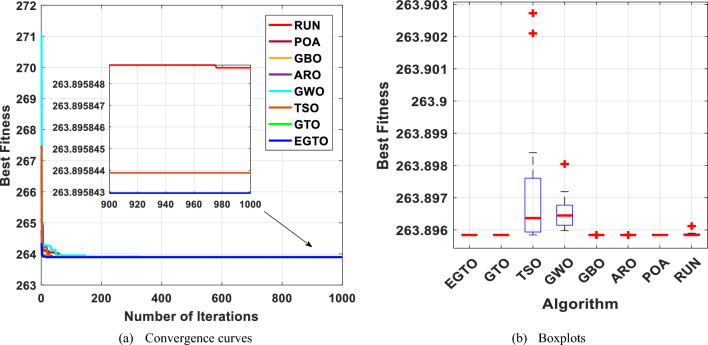
The convergence curves and boxplots of all algorithms for three-bar truss problem.

This optimization challenge is of great significance in civil engineering applications, as it seeks to achieve an efficient and lightweight design for the truss structure. Table 10 shows the performance of several algorithms, such as GTO, TSO, GWO, GBO, ARO, POA, RUN, and the proposed EGTO algorithm for solving the problem of the three-bar truss design problem. The results demonstrate that the EGTO algorithm indicates an outperformance compared to other algorithms. The minimum weight is 263.895, and the optimal values for × 1 and × 2 are 0.788675 and 0.408248, respectively. Figure 27 shows the convergence curves and the boxplot of these algorithms during solving the problem. Additionally, a comparison of the statistical results is given in Table 11, the mean and STD values obtained by the proposed EGTO, GTO, and POA algorithms are the best among all other counterparts.

**Table 10:** Results for the three-bar truss problem.

Algorithms	Optimal values for variables		Optimum cost
	x_1_	x_2_	
EGTO	0.78867513483	0.40824828109	263.89584294121
GTO	0.78867513483	0.40824828109	263.89584294121
TSO	0.788641813	0.408342532	263.89584387695
GWO	0.788829349	0.407813414	263.8959741587
GBO	0.788675135	0.40824828	263.89584294121
ARO	0.7886751295	0.4082482961	263.8958429412
POA	0.788675129	0.408248298	263.8958429
RUN	0.7886644263	0.4082785702	263.8958430255

**Table 11:** Statistical results for the three-bar truss problem.

Function	EGTO	GTO	TSO	GWO	GBO	ARO	POA	RUN
Best	**263.8958**	**263.8958**	263.8958	263.896	**263.8958**	**263.8958**	**263.8958**	**263.8958**
Mean	**263.8958**	**263.8958**	263.8972	263.8965	**263.8958**	**263.8958**	**263.8958**	263.8959
Median	**263.8958**	**263.8958**	263.8964	263.8964	**263.8958**	**263.8958**	**263.8958**	**263.8958**
Worst	**263.8958**	**263.8958**	263.9027	263.898	**263.8958**	**263.8958**	**263.8958**	263.8961
Std	**5.83E−14**	**5.83E−14**	0.001957	0.000504	1.14E−13	5.53E−14	5.83E−14	6.21E−05

Bold values have the best performance.

### Tension/compression spring design

**Figure 28:**
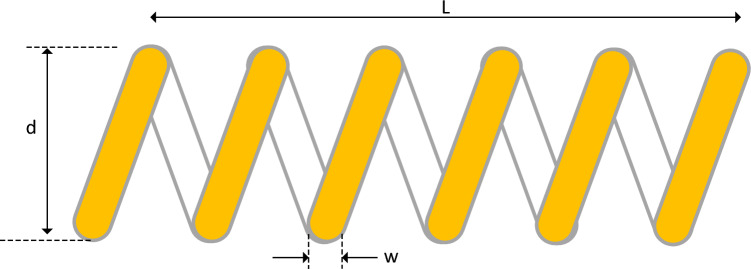
Schematic of tension/compression spring design problem.

We also investigated the Tension/Compression Spring Design (TCSD) problem as our second engineering optimization problem. The objective in this problem, as depicted in Fig. 28, is to minimize the weight of a tension/compression spring design^53^. The variables involved in this problem include the wire diameter (d), mean coil diameter (D), and the number of active coils (N). The problem is subject to constraints related to surge frequency, minimum deflection, and shear stress. The mathematical definitions for this problem are provided below. Furthermore, Table 12 presents the results obtained from applying the EGTO algorithm and other compared metaheuristic algorithms. Based on the results, the EGTO algorithm outperforms the other metaheuristic algorithms in terms of finding the optimal cost.

$$\begin{aligned} {\text{Consider}}:{ }\vec{x} & = \left[ {x_{1} x_{2} x_{3} } \right] = \left[ {d D N} \right], \\ {\text{Minimize }}f\left( {\vec{x}} \right) & = \left( {x_{3} + 2} \right)x_{2} x_{1}^{2} , \\ {\text{Subject to }}g_{1} \left( {\vec{x}} \right) & = 1 - \frac{{x_{2}^{3} x_{3} }}{{71785x_{1}^{4} }} \le 0, \\ g_{2} \left( {\vec{x}} \right) & = \frac{{4x_{2}^{2} - x_{1} x_{2} }}{{12566\left( {x_{2} x_{1}^{3} - x_{1}^{4} } \right)}} + \frac{1}{{5108x_{1}^{2} }} - 1 \le 0, \\ g_{3} \left( {\vec{x}} \right) & = 1 - \frac{{140.45x_{1} }}{{x_{2}^{2} x_{3} }} \le 0, \\ g_{4} \left( {\vec{x}} \right) & = \frac{{x_{1} + x_{2} }}{1.5} \le 0, \\ \end{aligned}$$

**Table 12:** Comparison results for the tension/compression spring design problem.

Algorithms	Optimal values for variables			Optimum cost
	d	D	P	
EGTO	0.051684027	0.356596637	11.29606918	0.012665233
GTO	0.051684027	0.356596637	11.29606918	0.012665233
TSO	0.051509705	0.352418212	11.54555779	0.012665821
GWO	0.051439916	0.350750504	11.64906365	0.012667813
GBO	0.051680283	0.356506591	11.30135554	0.012665234
ARO	0.051686854	0.356664511	11.29210027	0.012665248
POA	0.051798996	0.359368092	11.13526982	0.012665466
RUN	0.051356234	0.348763445	11.77100947	0.012667271

Variable range: $$0.05\le {x}_{1}\le 2.00$$, $$0.25\le {x}_{2}\le 1.30$$, $$2.00\le {x}_{3}\le 15.0$$.

The proposed EGTO's performance evaluation on this application is compared with that of TSO, GWO, GBO, ARO, POA, RUN, and GTO, as presented in Table 12. The results clearly indicate that EGTO outperforms all other comparison algorithms, yielding the minimum weight of 0.01266233 for the optimal solution ($\overrightarrow{x}$ = [0.051684027, 0.356596637, 11.29606918]). This remarkable achievement underscores the effectiveness of EGTO in efficiently resolving the tension/compression spring design problem. Its ability to find superior solutions showcases the notable merits and potential of EGTO in tackling such complex engineering optimization challenges.

**Figure 29:**
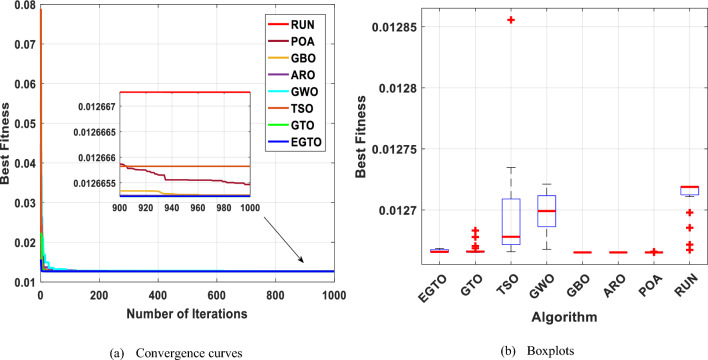
The convergence curves and boxplots of all algorithms for tension/compression spring problem.

Figure 29 displays the convergence curves and the boxplot of these techniques during solving the tension/compression spring design problem. Moreover, a comparison of the statistical results is presented in Table 13, the mean and STD values obtained by the proposed EGTO, ARO, GBO, and POA algorithms are the best among all other counterparts.

**Table 13:** Statistical results for the tension/compression spring design problem.

Function	EGTO	GTO	TSO	GWO	GBO	ARO	POA	RUN
Best	**0.012665**	**0.012665**	0.012666	0.012668	**0.012665**	**0.012665**	**0.012665**	0.012667
Mean	**0.012665**	0.012668	0.012695	0.012697	**0.012665**	**0.012665**	**0.012665**	0.01271
Median	0.012666	0.012666	0.012678	0.012699	**0.012665**	**0.012665**	**0.012665**	0.012719
Worst	0.012668	0.012683	0.012856	0.012721	**0.012665**	**0.012665**	0.012666	0.012719
Std	1.13E−06	4.67E−06	4.32E−05	1.59E−05	**4.08E−08**	5.49E−08	1.39E−07	1.65E−05

Bold values have the best performance.

### Pressure vessel design problem

Within this study, the 3rd optimization problem addressed is the Pressure Vessel Design (PVD) problem. The main objective of this problem is to decrease the overall cost associated with a cylindrical pressure vessel, encompassing material, welding, and forming costs^54^. The vessel consists of a hemispherical-shaped head with capped ends. The problem involves variables such as the head thickness (Th), the length of the cylindrical section excluding the head (L), the shell thickness (Ts), and the inner radius (R). Additionally, there are four constraints in this problem that can be expressed as follows.

$$\begin{gathered} {\text{Consider}}:\vec{x} = \left[ {x_{1} x_{2} x_{3} x_{4} } \right] = \left[ {T_{s} T_{h} RL} \right], \hfill \\ {\text{Minimize }}f\left( {\vec{x}} \right) = 0.6224x_{1} x_{3} x_{4} + 1.7781x_{2} x_{3}^{2} + 3.1661x_{1}^{2} x_{4} + 19.84x_{1}^{2} x_{3} , \hfill \\ {\text{Subject to }}g_{1} \left( {\vec{x}} \right) = - x_{1} + 0.0193x_{3} \le 0, \hfill \\ g_{2} \left( {\vec{x}} \right) = - x_{2} + 0.00954x_{3} \le 0, \hfill \\ g_{3} \left( {\vec{x}} \right) = - \pi x_{3}^{2} x_{4} - \frac{4}{3}\pi x_{3}^{3} + 1296000 \le 0, \hfill \\ g_{4} \left( {\vec{x}} \right) = x_{4} - 240 \le 0, \hfill \\ \end{gathered}$$

Variable range: $$0\le {x}_{1}\le 99$$, $$0\le {x}_{2}\le 99$$, $$10\le {x}_{3}\le 200$$, $$10\le {x}_{4}\le 200$$.

The pressure vessel design problem is optimized by the proposed EGTO technique, and the results are compared with those of popular traditional and recent algorithms. Table 14 shows the solutions of EGTO and other algorithms, namely, TSO, GWO, GBO, ARO, POA, RUN and conventional GTO algorithms. Based on the simulation results, the proposed algorithm exhibited superior performance in comparison to the other algorithms utilized in finding the optimal cost. BGWO is superior to all the other algorithms and obtains the best solution $\overrightarrow{x}$ = [0.778168279 0.384649018 40.31961872 200.0000], Minimize $f\left(\overrightarrow{x}\right)$ =5885.331251.

**Table 14:** Comparison results for the pressure vessel design problem.

Algorithms	Optimal values for variables				Optimum cost
	T_s_	T_h_	R	L	
EGTO	0.778168279	0.384649018	40.31961872	200	5885.331251
GTO	0.778168279	0.384649018	40.31961872	200	5885.331251
TSO	0.778168279	0.384649018	40.31961872	200	5885.331251
GWO	0.778264091	0.384705416	40.32161451	200	5886.514773
GBO	0.778168279	0.384649018	40.31961872	200	5885.331251
ARO	0.778168282	0.384649018	40.31961873	200	5885.331251
POA	0.778168279	0.384649018	40.31961872	200	5885.331251
RUN	0.778168537	0.384649215	40.31963894	199.9997508	5885.332783

**Figure 30:**
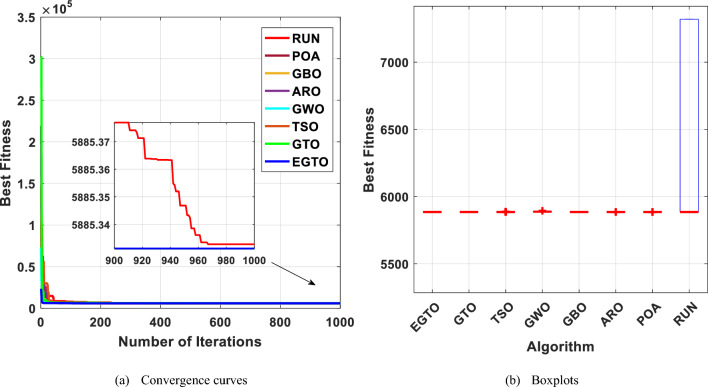
The convergence curves and boxplots of all techniques for pressure vessel design problem.

The results indicate that the proposed EGTO technique has strong optimization ability and excellent convergence. Figure 30 depicts the convergence curves and box plots of all algorithms while handling the problem. Table 15 also lists the statistical data, such as Best, Mean, Median, Worst, and STD. Therefore, it is determined that the reliability of the suggested EGTO algorithm is better for the pressure vessel design problem.

**Table 15:** Statistical results for the pressure vessel design problem.

Function	EGTO	GTO	TSO	GWO	GBO	ARO	POA	RUN
Best	**5885.331**	**5885.331**	**5885.331**	5886.515	**5885.331**	**5885.331**	**5885.331**	5885.333
Mean	**5885.331**	**5885.331**	5885.536	5888.73	**5885.331**	**5885.331**	**5885.331**	6315.424
Median	**5885.331**	**5885.331**	**5885.331**	5888.06	**5885.331**	**5885.331**	**5885.331**	5885.34
Worst	**5885.331**	**5885.331**	5888.008	5894.698	**5885.331**	**5885.331**	**5885.331**	7319.006
Std	1.43E−12	**1.34E−12**	0.623086	1.915396	1.34E−12	3.91E−06	1.76E−12	674.0292

Bold values have the best performance.

### Cantilever beam design problem

**Figure 31:**
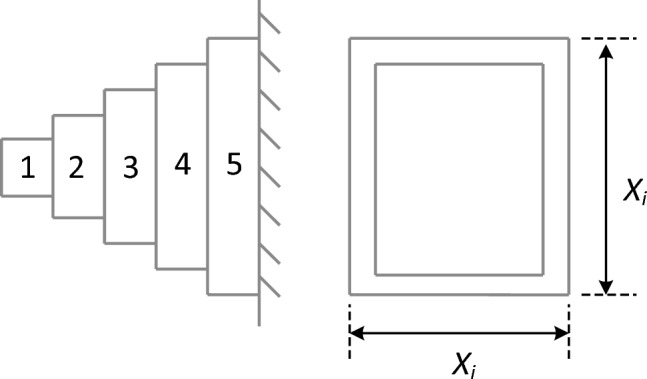
Schematic of Cantilever Beam Design problem.

The goal of the cantilever beam design (CBD) problem is to minimize the weight of a cantilever with five hollow blocks, as displayed in Fig. 31^54^. Therefore, five variables are involved, and the structural optimization problem can be expressed mathematically as:

$$\begin{aligned} {\text{Consider}}:\vec{x} & = \left[ {x_{1} x_{2} x_{3} x_{4} x_{5} } \right] \\ {\text{Minimize }}f\left( X \right) & = 0.0624\left( {x_{1} + x_{2} + x_{3} + x_{4} + x_{5} } \right) \\ {\text{Subject\, to\,}}G\left( X \right) & = \frac{61}{{x_{1}^{3} }} + \frac{37}{{x_{2}^{3} }} + \frac{19}{{x_{3}^{3} }} + \frac{7}{{x_{4}^{3} }} + \frac{1}{{x_{5}^{3} }} - 1 \le 0, \\ \end{aligned}$$

Variable range: $$0.01\le {x}_{i}\le 1.00; i\in 1,\dots .,5$$.

Table 16 lists the best solutions to this problem achieved by the EGTO method and the results are compared with those of recent algorithms. The results show that the EGTO algorithm effectively solves the problem and the design with the minimum weight $\overrightarrow{x}$ = [6.01606 5.309151 4.494265 3.501486 2.152698].

**Table 16:** The obtained results of the Cantilever Beam Design problem.

Algorithms	Optimal values for variables					Optimum cost
	${x}_{1}$	${x}_{2}$	${x}_{3}$	${x}_{4}$	${x}_{5}$	
EGTO	6.01606	5.309151	4.494265	3.501486	2.152698	1.339956361
GTO	6.016016	5.309174	4.49433	3.501475	2.152665	1.339956361
TSO	6.014715	5.309505	4.494716	3.502023	2.152701	1.339956416
GWO	6.016387	5.312571	4.496232	3.499537	2.148961	1.339958118
GBO	6.01606	5.309151	4.494265	3.501486	2.152698	1.339956361
ARO	6.016029	5.309241	4.493744	3.501819	2.152827	1.33995638
POA	6.016016	5.309174	4.49433	3.501475	2.152665	1.339956361
RUN	6.015499	5.309524	4.494606	3.501638	2.152394	1.339956379

**Figure 32:**
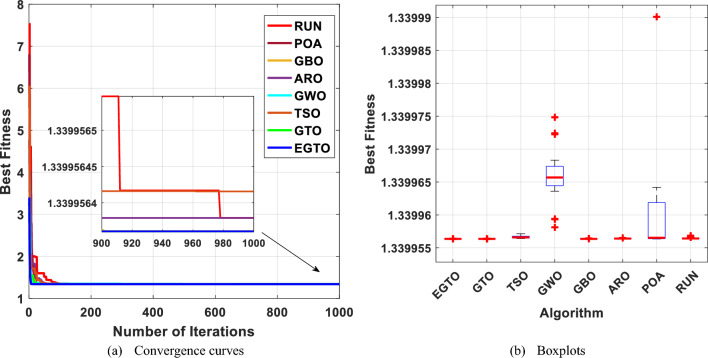
The convergence curves and boxplots of all techniques for Cantilever Beam Design problem.

Figure 32 depicts the convergence curves and box plots of all algorithms while handling the Cantilever Beam Design problem. Table 17 lists the statistical data, including Best, Mean, Median, Worst, and STD. Therefore, it is concluded that the reliability of the proposed EGTO algorithm is better for the problem.

**Table 17:** Statistical results for the Cantilever Beam Design problem.

Function	EGTO	GTO	TSO	GWO	GBO	ARO	POA	RUN
Best	**1.339956**	**1.339956**	**1.339956**	1.339958	**1.339956**	**1.339956**	**1.339956**	**1.339956**
Mean	**1.339956**	**1.339956**	1.339957	1.339966	**1.339956**	**1.339956**	1.33996	**1.339956**
Median	**1.339956**	**1.339956**	1.339957	1.339966	**1.339956**	**1.339956**	1.339957	**1.339956**
Worst	**1.339956**	**1.339956**	1.339957	1.339975	**1.339956**	1.339957	1.33999	1.339957
Std	**6.21E−09**	6.34E−09	2.13E−07	4.18E−06	1.62E−08	3.01E−08	7.58E−06	1.12E−07

Bold values have the best performance.

### Welded beam design

**Figure 33:**
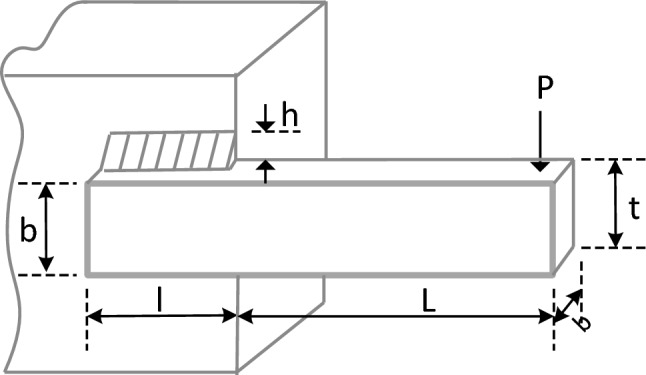
Schematic of welded beam design problem.

The objective of this particular engineering problem is to minimize the production cost of a welded beam^55^. Several optimization constraints need to be considered, including shear stress (τ), buckling load on the bar (Pc), end deflection of the beam (δ), and bending stress in the beam (Ѳ). The problem involves four variables: the thickness of the weld (h), the length of the attached part to the bar (l), the height of the bar (t), and the thickness of the bar (b) (refer to Fig. 33). Mathematically, the problem can be formulated as follows.

$$\begin{aligned} {\text{Consider}}:{ }\vec{x} & = \left[ {x_{1} x_{2} x_{3} x_{4} } \right] = \left[ {h l t b} \right], \\ {\text{Minimize }}f\left( {\vec{x}} \right) & = 1.1047x_{1}^{2} x_{2} + 0.04811x_{3} x_{4} \left( {14.0 + x_{2} } \right), \\ {\text{Subject to }}g_{1} \left( {\vec{x}} \right) & = \tau \left( {\vec{x}} \right) - \tau_{max} \le 0, \\ g_{2} \left( {\vec{x}} \right) & = \sigma \left( {\vec{x}} \right) - \sigma_{max} \le 0, \\ g_{3} \left( {\vec{x}} \right) & = \delta \left( {\vec{x}} \right) - \delta_{max} \le 0, \\ g_{4} \left( {\vec{x}} \right) & = x_{1} - x_{4} \le 0, \\ g_{5} \left( {\vec{x}} \right) & = p - p_{c} \left( {\vec{x}} \right) \le 0, \\ g_{6} \left( {\vec{x}} \right) & = 0.125 - x_{1} \le 0, \\ g_{7} \left( {\vec{x}} \right) & = 1.10471x_{1}^{2} x_{2} - 0.0481x_{3} x_{4} \left( {14.0 + x_{2} } \right) - 5.0 \le 0. \\ \end{aligned}$$

Variable range: $$0.1\le {x}_{1}\le 2.00$$, $$0.1\le {x}_{2}\le 10.0$$, $$0.1\le {x}_{3}\le 10.0$$, $$0.1\le {x}_{4}\le 2.00$$.

Table 18 displays the results attained from the analysis, demonstrating that the EGTO algorithm outperforms other metaheuristic algorithms by achieving a minimum cost design value. This fact shows that when the parameters are set h = 0.20572964, l = 3.470488666, t = 9.03662391, b = 0.20572964, the manufacturing cost of the welded beam design problem can reach 1.72485230859736. Therefore, the proposed EGTO algorithm has good potential in solving the welded beam design (WBD) problem.

**Table 18:** Results of the welded beam design problem.

Algorithms	Optimal values for variables				Optimum cost
	h	l	t	b	
EGTO	0.20572964	3.470488666	9.03662391	0.20572964	1.724852308597360
GTO	0.20572964	3.470488666	9.03662391	0.20572964	1.724852308597360
TSO	0.20572964	3.470488666	9.03662391	0.20572964	1.724852308605790
GWO	0.20561919	3.47268147	9.037531553	0.205740548	1.72521650544121
GBO	0.20572964	3.470488666	9.03662391	0.20572964	1.724852308597360
ARO	0.20573	3.470489	9.036624	0.20573	1.724852309
POA	0.20573	3.470489	9.036624	0.20573	1.724852309
RUN	0.20219	3.548344	9.036624	0.20573	1.729795448

**Figure 34:**
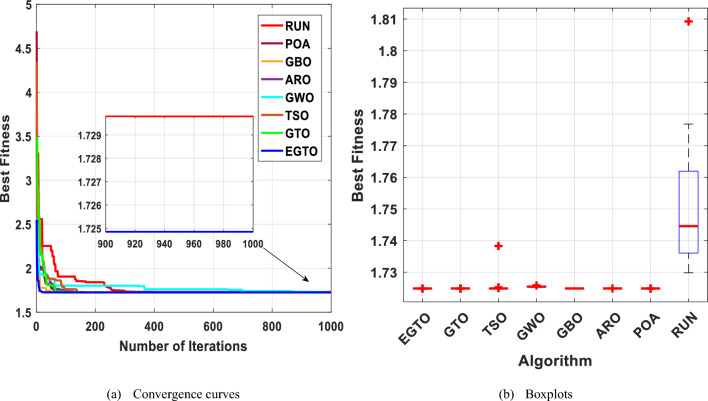
The convergence curves and boxplots of all techniques for welded beam design problem.

Figure 34 shows the convergence characteristics and box plots of all techniques for welded beam design problem. Table 19 presents the statistical data, including Best, Mean, Median, Worst, and STD. Therefore, it is concluded that the reliability of the proposed EGTO algorithm is better for the problem.

**Table 19:** Statistical results for the welded beam design problem.

Function	EGTO	GTO	TSO	GWO	GBO	ARO	POA	RUN
Best	**1.724852**	**1.724852**	**1.724852**	1.725217	**1.724852**	**1.724852**	**1.724852**	1.729795
Mean	**1.724852**	**1.724852**	1.725548	1.725449	**1.724852**	**1.724852**	**1.724852**	1.751008
Median	**1.724852**	**1.724852**	**1.724852**	1.725429	**1.724852**	**1.724852**	**1.724852**	1.744551
Worst	**1.724852**	**1.724852**	1.738277	1.725833	**1.724852**	**1.724852**	**1.724852**	1.809223
Std	**5.32E−16**	7.22E−16	0.002997	0.000163	6.83E−16	6.57E−15	3.12E−08	0.019885

Bold values have the best performance.

### Speed reducer design

**Figure 35:**
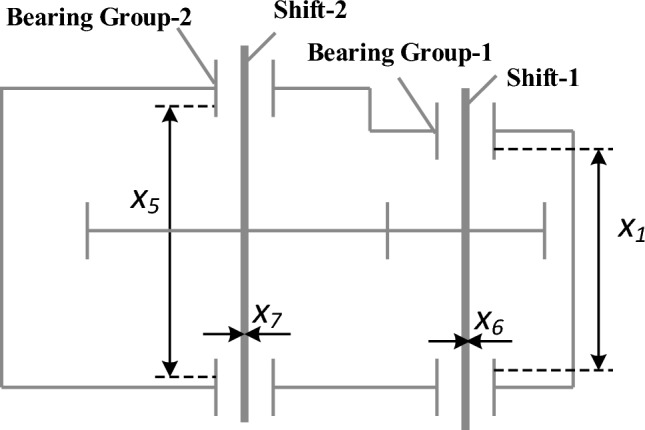
Schematic of speed reducer design problem.

This problem (Fig. 35) represents a well-known design challenge in mechanical systems. It involves optimizing the speed reducer, a crucial component of the gearbox, for various applications. The goal is to minimize 11 constraints associated with the weight of the speed reducer^56^. Among these constraints, seven are nonlinear, while the rest are linear inequalities. The four key parameters affecting the optimization process are as follows: bending stress of the gear teeth, surface stress, transverse deflections of the shafts, and stresses in the shafts. To solve this optimization problem, seven variables need to be considered: face width (b), module of teeth (m), the number of teeth in the pinion (z), length of the first shaft between bearings (l1), length of the second shaft between bearings (l2), diameter of the first shaft (d1), and diameter of the second shaft (d2). The equation representing this problem is as follows:

$${\text{Minimize\,}}f\left(b,m,z,{l}_{1},{l}_{2},{d}_{1},{d}_{2}\right)=0.7854{x}_{1}{x}_{2}^{2}\left(3.333{x}_{3}^{2}+14.9334{x}_{3}-43.0934\right)-1.508{x}_{1}\left({x}_{6}^{2}+{x}_{7}^{2}\right)+7.4777\left({x}_{6}^{3}+{x}_{7}^{3}\right)+0.7854\left({x}_{4}{x}_{6}^{2}+{{x}_{5}x}_{7}^{2}\right)$$

$$\mathrm{Subject\, to\,}{G}_{1}=\frac{27}{{x}_{1}{x}_{2}^{2}{x}_{3}}-1\le 0; {G}_{2}=\frac{397.5}{{x}_{1}{x}_{2}^{2}{x}_{3}^{2}}-1\le 0;$$

$${G}_{3}=\frac{1.93{x}_{4}^{3}}{{x}_{2}{x}_{6}^{4}{x}_{3}}-1\le 0; {G}_{4}=\frac{1.93{x}_{5}^{3}}{{x}_{2}{x}_{7}^{4}{x}_{3}}-1\le 0;$$

$${G}_{5}=\frac{\sqrt{{\left(\frac{745{x}_{4}}{{x}_{2}{x}_{3}}\right)}^{2}+16.9\times {10}^{6}}}{110{x}_{6}^{3}}-1\le 0; {G}_{6}=\frac{\sqrt{{\left(\frac{745{x}_{5}}{{x}_{2}{x}_{3}}\right)}^{2}+157.5\times {10}^{6}}}{85{x}_{7}^{3}}-1\le 0;$$

$${G}_{7}=\frac{{x}_{2}{x}_{3}}{40}-1\le 0; {G}_{8}=\frac{5{x}_{2}}{{x}_{1}}-1\le 0;{G}_{9}=\frac{{x}_{1}}{{12x}_{2}}-1\le 0;$$

$${G}_{10}=\frac{{1.5x}_{6}+1.9}{{x}_{4}}-1\le 0; {G}_{11}=\frac{1.1{x}_{7}+1.9}{{x}_{5}}-1\le 0;$$

Variable range: $$2.6\le {x}_{1}\le 3.6; 0.7\le {x}_{2}\le 0.8;17\le {x}_{3}\le 28;7.3\le {x}_{4};{x}_{5}\le 8.3;2.9\le {x}_{6}\le 3.9; 5\le {x}_{7}\le 5.5.$$

**Figure 36:**
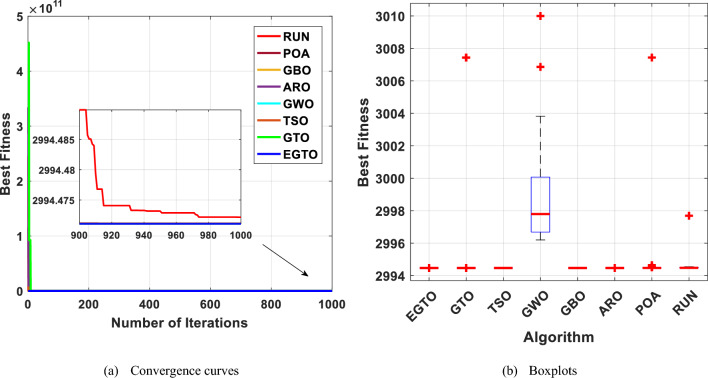
The convergence curves and boxplots of all algorithms for speed reducer design problem.

The attained results, which are shown in Table 20, prove that the EGTO technique finds a minimum cost design value in comparison to other metaheuristic algorithms. Figure 36 depicts the convergence curves and box plots of all algorithms while handling the problem. The proposed EGTO algorithm stands first in solving the speed reducer design (SRD) problem also. Table 21 lists the statistical results obtained by the EGTO and other selected algorithms. From Table 21, it can be seen that the EGTO performed better than all selected methods. Therefore, it is determined that the reliability of the suggested EGTO algorithm is better for the engineering design problem.

**Table 20:** Comparison results for speed reducer design problem.

Algorithms	Optimal values for variables							Optimum cost
	b	m	p	l_1_	l_2_	d_1_	d_2_	
EGTO	3.5	0.7	17	7.3	7.715319911	3.350214666	5.286654465	2994.471066
GTO	3.5	0.7	17	7.3	7.715319911	3.350214666	5.286654465	2994.471066
TSO	3.5	0.7	17	7.3	7.715319911	3.350214666	5.286654465	2994.471066
GWO	3.5	0.7	17	7.3	7.769914997	3.350599313	5.286742675	2996.19699
GBO	3.5	0.7	17	7.3	7.715319911	3.350214666	5.286654465	2994.471066
ARO	3.5	0.7	17	7.3	7.71532	3.350215	5.286654	2994.47107
POA	3.5	0.7	17	7.3	7.715321	3.350215	5.286655	2994.471146
RUN	3.5	0.7	17	7.3	7.71534	3.350217	5.286655	2994.472162

**Table 21:** Statistical results for speed reducer design problem.

Function	EGTO	GTO	TSO	GWO	GBO	ARO	POA	RUN
Best	**2994.471**	**2994.471**	**2994.471**	2996.197	**2994.471**	**2994.471**	**2994.471**	2994.472
Mean	**2994.471**	2996.416	**2994.471**	2999.336	**2994.471**	**2994.471**	2995.134	2994.651
Median	**2994.471**	**2994.471**	**2994.471**	2997.794	**2994.471**	**2994.471**	2994.472	2994.48
Worst	**2994.471**	3007.437	**2994.471**	3009.997	**2994.471**	**2994.471**	3007.437	2997.689
Std	**9.67E−13**	4.749871	8.01E−13	3.762576	9.33E−13	6.42E−11	2.896008	0.715362

Bold values have the best performance.

### Gear train design

As stated in Ref.^57^, the primary objective of optimizing the gear train design (GTD) is to achieve a gear transmission ratio as close as possible to 1/6.931. Let TA, TB, TD, and TF represent the number of teeth on gears A, B, D, and F, respectively. Since the number of teeth for each gear must be an integer between 12 and 60, the optimization problem is transformed into a constrained optimization problem with discrete variables. The formulation of the optimization problem is as follows:

$$\mathrm{Minimize\, }f={\left(\frac{1}{6.931}-\frac{{T}_{D}{T}_{B}}{{T}_{A}{T}_{F}}\right)}^{2}$$

Variable range: $$12\le {T}_{A}\le 60;12\le {T}_{B}\le 60;12\le {T}_{D}\le 60;12\le {T}_{F}\le 60$$.

The optimal solutions found by EGTO and other techniques are presented in Table 22. From this Table 22, it is seen that the results obtained using the proposed EGTO technique are the best, the optimal value is 0, GWO gets the worst results. The results display that the EGTO algorithm efficiently solves the problem and the design with the minimum fitness $\overrightarrow{x}$ = [59.10865551 35.44113483 13.11941687 54.52139629]. These experimental results present that the proposed EGTO technique has a strong exploitation.

**Table 22:** Results of the Gear train design problem.

Algorithms	Optimal values for variables				Optimum cost
	${T}_{A}$	${T}_{B}$	${T}_{D}$	${T}_{F}$	
EGTO	59.10865551	35.44113483	13.11941687	54.52139629	0
GTO	49.59351278	24.9585507	13.69391961	47.76592194	0
TSO	59.99999629	12	43.16138541	59.83031616	0
GWO	53.95860993	12	22.16042143	34.15815629	2.19246E−17
GBO	58.64588098	41.95268373	12.05514903	59.7710267	2.18986E−25
ARO	55.78268	19.04683	17.77422	42.06392	4.5135E−21
POA	47.4056	17.20186	12.59847	31.68542	0
RUN	55.32222	12.0058	32.81681	49.36097	0

**Figure 37:**
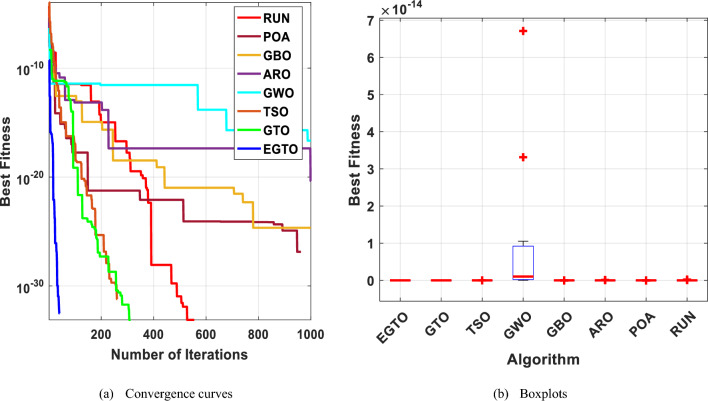
The convergence curves and boxplots of all algorithms for Gear train design problem.

The convergence curves and boxplots of the gear train design problem using the EGTO and other algorithms are shown in Fig. 37. The proposed EGTO algorithm is evaluated and compared with several other optimization algorithms, including GTO, TSO, GWO, GBO, ARO, POA, and RUN. The evaluation is conducted for the Gear Train Design Problem. Table 23 presents the statistical results of these algorithms based on their performance in solving the Gear Train Design Problem. The metrics used to measure the performance include "Best," "Mean," "Median," "Worst," and "Std" for each algorithm. The EGTO algorithm achieved the best result with a fitness value of 0, indicating its ability to find an optimal solution for the Gear Train Design Problem. Also, The EGTO algorithm achieved an average fitness value of 0, indicating that, on average, it performed exceptionally well in finding solutions close to the optimal value. The EGTO algorithm achieved a very low standard deviation of 0, indicating its consistent performance and stability in finding near-optimal solutions. Overall, based on the statistical results, the proposed EGTO algorithm demonstrates exceptional performance in solving the Gear Train Design Problem compared to the other evaluated algorithms. Its ability to consistently find near-optimal solutions and its low variability make it a promising choice for similar constrained optimization problems.

**Table 23:** Statistical results for Gear train design problem.

Function	EGTO	GTO	TSO	GWO	GBO	ARO	POA	RUN
Best	**0**	**0**	**0**	2.19E−17	2.19E−25	4.51E−21	**0**	**0**
Mean	**0**	**0**	3.91E−27	8.14E−15	5.57E−21	1.13E−17	1.58E−24	2.46E−17
Median	**0**	**0**	**0**	1.02E−15	4.68E−22	9.36E−19	3.53E−27	1.94E−18
Worst	**0**	**0**	7.81E−26	6.71E−14	5.42E−20	1.22E−16	2.69E−23	2.12E−16
Std	**0**	**0**	1.75E−26	1.59E−14	1.31E−20	2.99E−17	5.99E−24	5.45E−17

Bold values have the best performance.

### Wilcoxon rank-sum test results

In this subsection, the variances between EGTO and other techniques are additional analyzed statistically using the Wilcoxon rank-sum test (WRST), which is a paired assessment is employed to notice significant differences between the two algorithms. The attained results of the test between EGTO and each algorithm, conducted at a significance level of α = 0.05 are presented in Table 24, where the symbols "+/=/−" show whether EGTO performs better, similarly, or worse than the compared technique. Additionally, the table includes statistical findings for EGTO across different dimensions and functions, signifying whether EGTO performs better, similarly, or worse than the comparison algorithm. EGTO demonstrates superior statistical performance in seven real-world constrained engineering design problems including Three-Bar Truss Design, Compression Spring Design, Pressure Vessel Design, Cantilever Beam Design, Welded Beam Design, Speed Reducer Design, and Gear Train Design when compared to other techniques, affirming its significant dominance across most functions. Consequently, it is confidently concluded that the proposed EGTO technique exhibits the best overall performance when compared to other methods.

**Table 24:** Statistical results of Wilcoxon rank-sum test.

EGTO vs	GTO		TSO		GWO		POA		ARO		GBO		RUN	
Problem	P	Winner	P	Winner	P	Winner	P	Winner	P	Winner	P	Winner	P	Winner
3BTD	NaN	=	8.01E−09	+	8.01E−09	+	8.01E−09	+	8.01E−09	+	5.50E−09	+	8.01E−09	+
TSCD	6.55E−01	+	7.95E−07	+	1.06E−07	+	6.80E−08	+	1.03E−06	+	1.12E−03	−	1.01E−03	+
PVD	5.34E−01	=	3.42E−07	+	4.14E−08	+	4.14E−08	+	4.14E−08	+	5.34E−01	=	4.14E−08	+
CBD	6.75E−01	=	6.78E−08	+	6.78E−08	+	6.78E−08	+	9.15E−08	+	9.20E−04	+	7.88E−08	+
WBD	6.52E−01	=	2.43E−08	+	2.43E−08	+	2.43E−08	+	2.32E−03	+	4.00E−02	+	1.30E−03	+
SRD	2.04E−01	+	1.37E−02	+	1.95E−08	+	1.95E−08	+	7.54E−04	+	5.74E−01	=	1.95E−08	+
GTD	NaN	=	3.42E−01	=	8.01E−09	+	8.01E−09	+	NaN	=	8.01E−09	+	NaN	=
WRST (+/=/−)	2/5/0		6/1/0		7/0/0		7/0/0		6/1/0		4/2/1		6/1/0	

### Friedman’s rank test results

Table 25 shows the statistical results achieved using Friedman tests^58^ for seven real-world constrained engineering design problems using the studied algorithms. A lower ranking value indicates superior algorithm performance. According to the results, the ranking order of the six techniques is as follows: EGTO, GTO, GBO, RUN, ARO, TSO, GWO, and POA.

**Table 25:** Friedman test for the eight algorithms.

Problem	EGTO	GTO	TSO	GWO	POA	ARO	GBO	RUN
3BTD	1.5	1.5	6.6	6.6	7.7	4.45	3	4.65
TSCD	2.6	2.85	5.5	6.65	7	6.3	1.35	3.75
PVD	1.925	2.125	4.05	6	7.5	7.25	2.1	5.05
CBD	1.65	1.7	5.65	7	8	4.55	2.7	4.75
WBD	2.625	2.425	6.1	6.95	7.95	3.8	2.15	4
SRD	2.475	3.45	3.2	6.9	7.95	3.725	2.5	5.8
GTD	2.975	2.975	3.1	7.45	7.55	2.975	6	2.975
Mean ranks	2.25	2.432143	4.885714	6.792857	7.664286	4.721429	2.828571	4.425

**Figure 38:**
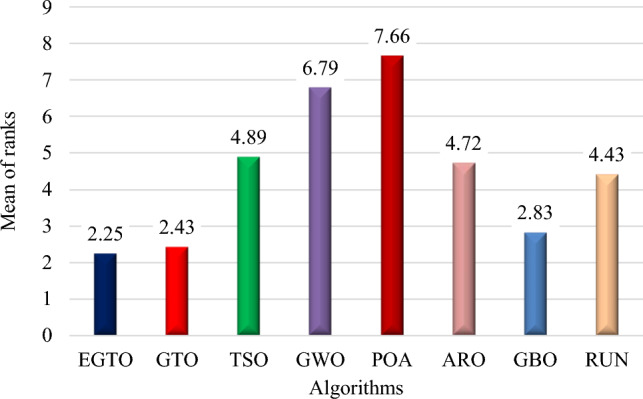
Mean ranks achieved using Friedman’s rank test for the seven real-world constrained engineering design problems using various algorithms.

Furthermore, Fig. 38 presents the mean ranks obtained from Friedman's rank test for the seven real-world constrained engineering design problems using various algorithms. This visualization provides a clear comparison of the algorithms' performances across the cases, helping to identify any significant differences in their ranks. The top-ranking position clearly indicates that EGTO is the most effective algorithm among the eight considered.

## Conclusion

In this article, an enhanced GTO technique, namely EGTO, has been proposed to effectively and precisely improve the performance of the GTO technique. The EGTO technique has been  tested on a set of 23 benchmark functions with different dimensions to evaluate the exploration and exploitation phases for avoiding local optimum. Initially, the results reveal that the EGTO algorithm is the best among these well-known algorithms such as Tuna Swarm Optimization (TSO), Grey Wolf optimizer (GWO) algorithm, Gradient-based optimizer (GBO) algorithm, Artificial rabbits optimization (ARO), Pelican Optimization Algorithm (POA), and RUN algorithm. The EGTO algorithm has been tested on thirty-one benchmark functions to analyze the exploration, exploitation, local optima avoidance, and convergence behavior. The results of these test functions show that the EGTO algorithm is the best optimizer which provides very competitive results as compared to other optimizers. The statistical testing has been carried out to demonstrate the superiority of the EGTO algorithm compared to other metaheuristics. Also, to validate the effectiveness of the proposed EGTO in solving highly difficult and complex composition functions, and providing a rigorous assessment of the algorithms, the CEC2019 functions were used in this paper, and the results of the proposed algorithm were compared with the results of several optimization algorithms. The simulation results and statistical analysis reveal that EGTO is capable of attaining the optimal solutions for maximum benchmark functions while maintaining a good balance between exploration and exploitation. In addition, the EGTO algorithm has been employed for seven real-world constrained engineering design problems (i.e., Three-Bar Truss Design, Compression Spring Design, Pressure Vessel Design, Cantilever Beam Design, Welded Beam Design, Speed Reducer Design, and Gear Train Design) which demonstrates that the EGTO algorithm has high-performance capability in unknown search spaces. This paper puts forward several research directions in the EGTO algorithm that may be applied to solve multi-objective optimization problems in future work. Also, Binary and multi-objective versions of the EGTO algorithm can be seen as an interesting direction for future contribution.

### Supplementary Information

Supplementary Information.

## Data Availability

The datasets generated during and/or analyzed during the current study are available from the corresponding author on reasonable request.
