# Supplementary material for: Enhanced gorilla troops optimizer powered by marine predator algorithm: global optimization and engineering design
Source: Sci Rep. 2024 Apr 1;14:7650. doi: 10.1038/s41598-024-57098-8 (PMC10985116; doi:10.1038/s41598-024-57098-8)
Supplement: Supplementary file 1 — Supplementary Information. [file 41598_2024_57098_MOESM1_ESM.docx]

Supplementary Material

Table A Benchmark functions

| Function | Dim | Range | f_min_ |
| --- | --- | --- | --- |
| $F_{1}\left( x \right)=\sum_{i=1}^{N} x_{i}^{2}$ | 30, 50, 100 | [-100,100] | 0 |
| $F_{2}\left( x \right)=\sum_{i=1}^{N} \left\vert x_{i} \right\vert+\prod_{i=1}^{N} \left\vert x_{i} \right\vert$ | 30, 50, 100 | [-10,10] | 0 |
| $F_{3}\left( x \right)=\sum_{i=1}^{N} \left( \sum_{j-1}^{i} x_{j} \right)^{2}$ | 30, 50, 100 | [-100,100] | 0 |
| $F_{4}\left( x \right)={max}_{i}\left\{ \left\vert x_{i} \right\vert,1\leq i\leq N \right\}$ | 30, 50, 100 | [-100,100] | 0 |
| $F_{5}\left( x \right)=\sum_{i=1}^{N-1} [100\left( x_{i+1}-x_{i}^{2} \right)^{2}+\left( x_{i}-1 \right)^{2}$ | 30, 50, 100 | [-30,30] | 0 |
| $F_{6}\left( x \right)=\sum_{i=1}^{N} \left( \left\vert x_{i}+0.5 \right\vert\right)^{2}$ | 30, 50, 100 | [-100,100] | 0 |
| $F_{7}\left( x \right)=\sum_{i=1}^{N} {ix}_{i}^{4}+random[0,1]$ | 30, 50, 100 | [-1.28,1.28] | 0 |
| $F_{8}\left( x \right)=\sum_{i=1}^{N} -x_{i}\sin\left( \sqrt{\left\vert x_{i} \right\vert} \right)$ | 30, 50, 100 | [-500,500] | -418.9829×dim |
| $F_{9}\left( x \right)=\sum_{i=1}^{N} [x_{i}^{2}-10\cos\left( 2\pi x_{i} \right) +10]$ | 30, 50, 100 | [-5.12,5.12] | 0 |
| $F_{10}\left( x \right)=-20\exp\left( -0.2\sqrt{\frac{1}{N}\sum_{i=1}^{N} x_{i}^{2}} \right)-exp\left( \frac{1}{N}\sum_{i=1}^{N} \cos\left( 2\pi x_{i} \right) \right)+20+e$ | 30, 50, 100 | [-32,32] | 0 |
| $F_{11}\left( x \right)=\frac{1}{4000}\sum_{i=1}^{N} x_{i}^{2}-\prod_{i=1}^{N} \cos\left( \frac{x_{i}}{\sqrt{i}} \right)+1$ | 30, 50, 100 | [-600,600] | 0 |
| $F_{12}\left( x \right)=\frac{\pi}{n}\left\{ 10\sin\left( \pi y_{1} \right)+\sum_{i=1}^{n-1} \left( y_{i}-1 \right)^{2}\left[ 1+10\sin^{2} \left( \pi y_{i+1} \right) \right]+\left( y_{n}-1 \right)^{2} \right\}+\sum_{i=1}^{n} u\left( x_{i},10,100,4 \right)$  $y_{i}=1+\frac{x_{i}+1}{4}u\left( x_{i},a,k,m \right)=\left\{ \begin{aligned} k\left( x_{i}-a \right)^{m} x_{i}>a \\ 0 -a<x_{i}<a \\ k\left( {-x}_{i}-a \right)^{m} x_{i}<-a \end{aligned} \right.$ | 30, 50, 100 | [-50,50] | 0 |
| $F_{13}\left( x \right)=0.1\left\{ \sin^{2} \left( 3\pi x_{1} \right)+\sum_{i=1}^{n} \left( x_{i}-1 \right)^{2}\left[ 1+\sin^{2} \left( \pi x_{i}+1 \right) \right]+\left( x_{n}-1 \right)^{2}\left[ 1+\sin^{2} \left( 2\pi x_{n} \right) \right] \right\}+\sum_{i=1}^{n} u\left( x_{i},5,100,4 \right)$ | 30, 50, 100 | [-50,50] | 0 |
| $F_{14}\left( x \right)=\left( \frac{1}{500}+\sum_{j=1}^{25} \frac{1}{j+\sum_{i=1}^{2} \left( x_{i}-a_{ij} \right)^{6}} \right)^{-1}$ | 2 | [-65,65] | 1 |
| $F_{15}\left( x \right)=\sum_{i=1}^{11} \left[ a_{i}-\frac{x_{1}\left( b_{i}^{2}+b_{i}x_{2} \right)}{b_{i}^{2}+b_{i}x_{3}+x_{4}} \right]^{2}$ | 4 | [-5,5] | 0.00030 |
| $F_{16}\left( x \right)=4x_{1}^{2}-2.1x_{1}^{4}+\frac{1}{3}x_{1}^{6}+x_{1}x_{2}-4x_{2}^{2}+4x_{2}^{4}$ | 2 | [-5,5] | -1.0316 |
| $F_{17}\left( x \right)=\left( x_{2}-\frac{5.1}{4\pi^{2}}x_{1}^{2}+\frac{5}{\pi}x_{1}-6 \right)+10\left( 1-\frac{1}{8\pi} \right)\cos x_{1}+10$ | 2 | [-5,5] | 0.398 |
| $F_{18}\left( x \right)=\left[ 1+\left( x_{1}+x_{2}+1 \right)^{2}\left( 19-14x_{1}+3x_{1}^{2}-14x_{2}+6x_{1}x_{2}+3x_{2}^{2} \right) \right]\times\left[ 30+\left( 2x_{1}-3x_{2} \right)^{2}\times\left( 18-32x_{1}+12x_{1}^{2}+48x_{2}-36x_{1}x_{2}+27x_{2}^{2} \right) \right]$ | 2 | [-2,2] | 3 |
| $F_{19}\left( x \right)=-\sum_{i=1}^{4} c_{i} exp\left( -\sum_{j=1}^{3} a_{ij}\left( x_{j}-p_{ij} \right)^{2} \right)$ | 3 | [1,3] | -3.86 |
| $F_{20}\left( x \right)=-\sum_{i=1}^{4} c_{i} exp\left( -\sum_{j=1}^{6} a_{ij}\left( x_{j}-p_{ij} \right)^{2} \right)$ | 6 | [0,1] | -3.32 |
| $F_{21}\left( x \right)=-\sum_{i=1}^{5} \left[ \left( X-a_{i} \right)\left( X-a_{i} \right)^{T}+c_{i} \right]^{-1}$ | 4 | [0,10] | -10.1532 |
| $F_{22}\left( x \right)=-\sum_{i=1}^{7} \left[ \left( X-a_{i} \right)\left( X-a_{i} \right)^{T}+c_{i} \right]^{-1}$ | 4 | [0,10] | -10.4028 |
| $F_{23}\left( x \right)=-\sum_{i=1}^{10} \left[ \left( X-a_{i} \right)\left( X-a_{i} \right)^{T}+c_{i} \right]^{-1}$ | 4 | [0,10] | -10.5363 |

Table B Modern 10 benchmark test functions from CEC2019

| No | Functions | Dimension | Range | fmin |
| --- | --- | --- | --- | --- |
| CEC01 | Storn’s Chebyshev polynomial fitting problem | 9 | [- 8192,8192] | 1 |
| CEC02 | Inverse Hilbert matrix problem | 16 | [- 16,384,16384] | 1 |
| CEC03 | Lennard–Jones minimum energy cluster | 18 | [- 4, 4] | 1 |
| CEC04 | Rastrigin’s function | 10 | [- 100,100] | 1 |
| CEC05 | Griewangk’s function | 10 | [- 100,100] | 1 |
| CEC06 | Weierstrass function | 10 | [- 100,100] | 1 |
| CEC07 | Modified Schwefel’s function | 10 | [- 100,100] | 1 |
| CEC08 | Expanded Schaffer’s F6 function | 10 | [- 100,100] | 1 |
| CEC09 | Happy cat function | 10 | [- 100,100] | 1 |
| CEC10 | Ackley function | 10 | [- 100,100] | 1 |

MATLAB codes of all competitive techniques used in this paper can be obtained from the following links:

GWO: <https://seyedalimirjalili.com/gwo>

MPA: <https://www.mathworks.com/matlabcentral/fileexchange/74578-marine-predators-algorithm-mpa>

GTO: <https://www.mathworks.com/matlabcentral/fileexchange/95953-artificial-gorilla-troops-optimizer?s_tid=FX_rc1_behav>

TSO: <https://www.mathworks.com/matlabcentral/fileexchange/101734-tuna-swarm-optimization>

GBO: <https://www.mathworks.com/matlabcentral/fileexchange/131588-gradient-based-optimizer>

ARO: <https://www.mathworks.com/matlabcentral/fileexchange/110250-artificial-rabbits-optimization-aro>

RUN: <https://www.mathworks.com/matlabcentral/fileexchange/91075-run-beyond-metaphor-an-efficient-optimization-algorithm>
